# Activation of G_s_ signaling in mouse enteroendocrine K cells greatly improves obesity- and diabetes-related metabolic deficits

**DOI:** 10.1172/JCI182325

**Published:** 2024-10-22

**Authors:** Antwi-Boasiako Oteng, Liu Liu, Yinghong Cui, Oksana Gavrilova, Huiyan Lu, Min Chen, Lee S. Weinstein, Jonathan E. Campbell, Jo E. Lewis, Fiona M. Gribble, Frank Reimann, Jürgen Wess

**Affiliations:** 1 Molecular Signaling Section, Laboratory of Bioorganic Chemistry, National Institute of Diabetes and Digestive and Kidney Diseases (NIDDK), NIH, Bethesda, Maryland, USA.; 2 Center for Research on Genomics and Global Health (CRGGH), National Human Genome Research Institute (NHGRI), NIH, Bethesda, Maryland, USA.; 3 Mouse Metabolism Core,; 4 Mouse Transgenic Core Facility, NIDDK, NIH, Bethesda, Maryland, USA.; 5 Signal Transduction Section, Metabolic Diseases Branch, NIDDK, NIH, Bethesda, Maryland, USA.; 6 Duke Molecular Physiology Institute, Duke University, Durham, North Carolina, USA.; 7 MRC Metabolic Diseases Unit, Institute of Metabolic Science, University of Cambridge, Addenbrooke’s Hospital, Cambridge, United Kingdom.

**Keywords:** Endocrinology, Diabetes, G protein&ndash;coupled receptors, G proteins

## Abstract

Following a meal, glucagon-like peptide 1 (GLP1) and glucose-dependent insulinotropic polypeptide (GIP), the 2 major incretins promoting insulin release, are secreted from specialized enteroendocrine cells (L and K cells, respectively). Although GIP is the dominant incretin in humans, the detailed molecular mechanisms governing its release remain to be explored. GIP secretion is regulated by the activity of G protein–coupled receptors (GPCRs) expressed by K cells. GPCRs couple to 1 or more specific classes of heterotrimeric G proteins. In the present study, we focused on the potential metabolic roles of K cell G_s_. First, we generated a mouse model that allowed us to selectively stimulate K cell G_s_ signaling. Second, we generated a mouse strain harboring an inactivating mutation of *Gnas*, the gene encoding the α-subunit of G_s_, selectively in K cells. Metabolic phenotyping studies showed that acute or chronic stimulation of K cell G_s_ signaling greatly improved impaired glucose homeostasis in obese mice and in a mouse model of type 2 diabetes, due to enhanced GIP secretion. In contrast, K cell–specific *Gnas*-KO mice displayed markedly reduced plasma GIP levels. These data strongly suggest that strategies aimed at enhancing K cell G_s_ signaling may prove useful for the treatment of diabetes and related metabolic diseases.

## Abstract 2

We demonstrate that selective activation of G_s_ signaling in K-cells greatly ameliorates metabolic deficits in obese and diabetic mice due to enhanced GIP secretion.

## Graphical abstract

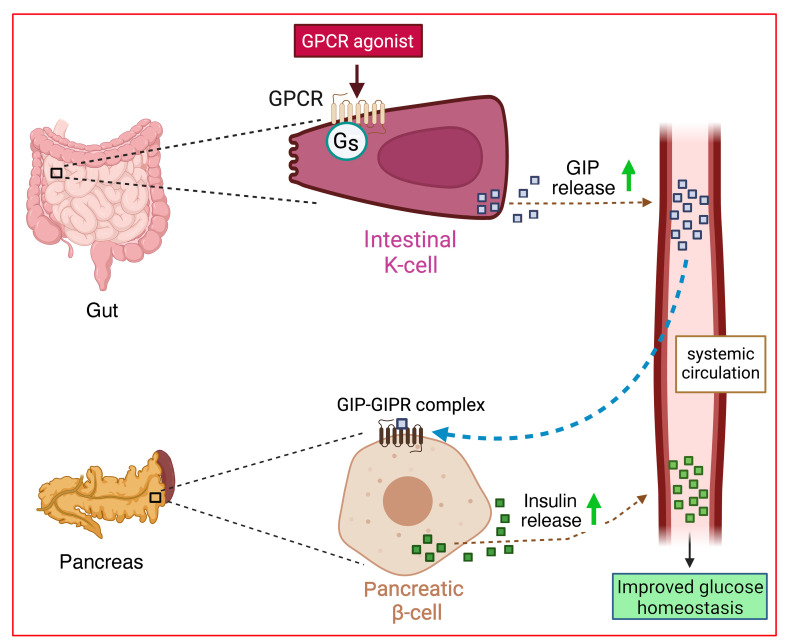

## Introduction

Incretins are polypeptide hormones that are released from specialized enteroendocrine cells after the intake of food (1–4). Numerous studies have shown that these intestinal peptide hormones play key roles in the maintenance of proper glucose homeostasis and various other important metabolic functions (1–4). The 2 major incretins are glucagon-like peptide 1 (GLP1) and glucose-dependent insulinotropic polypeptide (GIP) which act on pancreatic β cells to promote the release of insulin, thus contributing to the restoration of euglycemia after a meal (1–4). Besides β cells, both incretins can also modulate the activity of several other cell types, including specific neuronal subpopulations (4–6). The physiological actions of GLP1 and GIP are mediated by GLP1 and GIP receptors, respectively, which belong to the subfamily of class B GPCRs (7).

The recent development of highly selective GIP and GLP1 receptor antagonists has made it possible to study the relative contribution of endogenous GIP and GLP1 to enhanced insulin release and improved glucose homeostasis (3, 8). Studies with these agents have shown that the beneficial metabolic effects of GIP and GLP1 are additive, and that GIP is quantitatively more important than GLP1 in promoting insulin release and affecting postprandial glucose excursions, at least in healthy individuals (3, 8).

During the past decades, much research has focused on agents that can selectively stimulate GLP1 receptors for therapeutic purposes. These studies have led to the development of several clinically approved GLP1 receptor agonists that are highly efficacious in the treatment of type 2 diabetes (T2D) and obesity (9, 10). In contrast, research in the GIP field has been held back by initial findings that patients with T2D failed to release significant amounts of insulin following exogenously administered GIP (reviewed in ref. 11). Moreover, studies with GIP and GIP receptor–mutant mice and GIP receptor agonists and antagonists resulted in seemingly contradictory results (12, 13). Early work demonstrated that GIP receptor and GIP-KO mice showed reduced body weight gain under different experimental conditions, associated with improvements in glucose homeostasis (14, 15). The potential role of GIP receptor signaling to the regulation of body weight is also consistent with the outcome of a more recent human GWAS (16). These findings stimulated efforts to develop GIP receptor blockers as appetite-suppressive and antidiabetic drugs (reviewed in ref. 17). On the other hand, more recent studies showed that GIP receptor agonists, particularly in combination with GLP1 receptor agonists, are highly efficacious in reducing body weight and improving glucose homeostasis (reviewed in ref. 17). For example, treatment with tirzepatide, an FDA-approved dual GIP/GLP1 receptor agonist, causes pronounced reductions in body weight and striking improvements in blood glucose control (18, 19). Clinical trials demonstrated that tirzepatide administration resulted in more robust weight loss and improved glycemic control as compared with treatment with GLP1 receptor agonists alone (18, 19). The cellular and molecular mechanisms underlying the observation that both activation and blockade of GIP receptors can lead to similar metabolic outcomes are currently the matter of intense investigation.

Højberg et al. (20) demonstrated that the insulinotropic activity of GIP can be restored in T2D, at least partially, after drug-induced lowering of blood glucose levels. Moreover, a recent study in which diabetic individuals were treated with a selective GIP receptor antagonist indicated that endogenous GIP retains considerable efficacy in promoting insulin release in T2D (21).

Prompted by these recent studies, we tested the hypothesis that strategies aimed at enhancing the release of endogenous GIP from K cells might prove beneficial for the treatment of T2D and obesity-related metabolic disorders. Like other cell types, K cells express numerous GPCRs that are linked to different functional classes of heterotrimeric G proteins (G_q/11_, G_s_, G_i/o_, and G_12/13_) (6, 22). As a general rule, a specific GPCR is expressed in multiple tissues and cell types (23). For this reason, it has not been possible to explore the in vivo metabolic consequences of selective modulation of GIP release from enteroendocrine K cells by traditional pharmacological techniques. To overcome this obstacle, we took advantage of the availability of designer GPCRs known as designer receptors exclusively activated by designer drugs (DREADDs) (24–27). Following the recent development of a G_12_-coupled DREADD (28), DREADDs that are selectively linked to each of the 4 major G protein families are now available as powerful chemogenetic tools (27, 29). DREADDs can be selectively activated by synthetic drugs such as clozapine-*N*-oxide (CNO) (24) or deschloroclozapine (DCZ) (30, 31), which are otherwise pharmacologically inert, at least when used in the proper dose range (24, 27, 30).

In the present study, we examined whether selective activation of G_s_ signaling in mouse K cells was able to promote GIP release in vivo and, if so, how G_s_-mediated GIP secretion affects impaired glucose homeostasis in obese mice and in mice with partial ablation of β cells mimicking human T2D. To address these questions, we generated a DREADD mouse strain that selectively expressed a G_s_ DREADD (GsD) (25) in mouse K cells and subjected the resulting mutant mice to systematic metabolic studies. To complement this work, we also generated and analyzed another mouse model that lacked functional Gα_s_ selectively in K cells.

We found that chemogenetic activation of G_s_ signaling in K cells led to greatly improved glucose homeostasis in obese, glucose-intolerant mice and in a mouse model of T2D, most likely due to increased plasma GIP levels. In contrast, mice lacking functional Gα_s_ selectively in K cells showed reduced plasma GIP levels. Our data suggest that agents capable of promoting G_s_ signaling in K cells (e.g., agonists acting on G_s_-coupled receptors endogenously expressed by K cells) may emerge as drugs useful for the treatment of T2D and related metabolic disorders.

## Results

### Generation and functional characterization of K-GsD mice.

**Figure 1:**
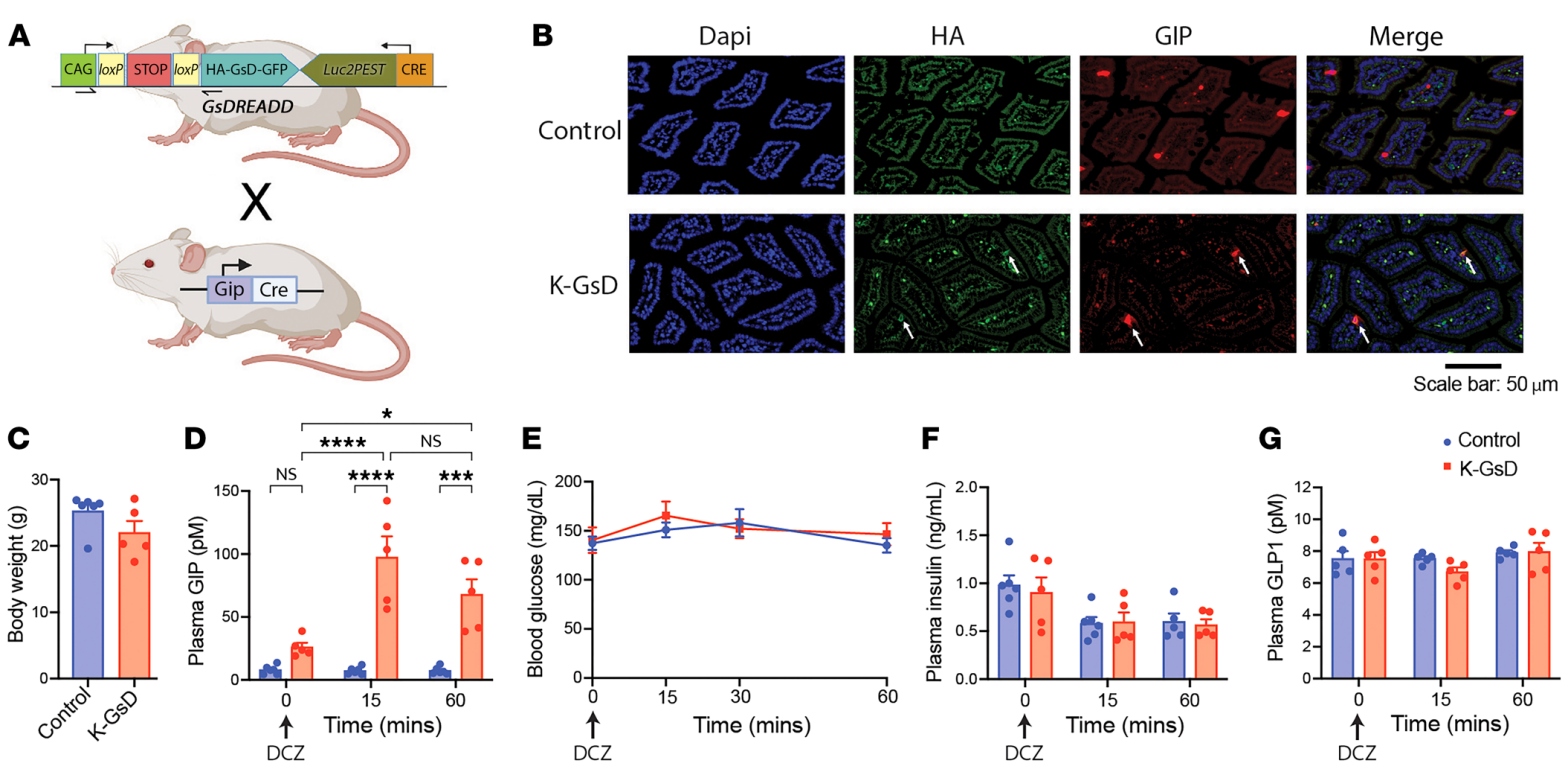
Selective activation of the GsD designer receptor in K cells stimulates GIP secretion in vivo. (**A**) Schematic depicting the generation of mice selectively expressing the GsD DREADD in K cells. (**B**) Representative immunohistochemical images showing colocalization of GIP and GsD (detected with an anti-HA antibody directed against the HA tag fused to the N-terminus of GsD) in the duodenal epithelium of K-GsD mice but not control littermates. White arrows point to individual K cells expressing GsD. Note that the anti-HA antibody caused marked nonspecific staining (staining seen in both control and K-GsD mice).Scale bar: 50 μm. (**C**) Body weight of 8-week-old K-GsD mice and control littermates consuming regular chow. (**D**–**G**) Treatment of K-GsD mice and control littermates with a single oral dose of DCZ (10 μg/kg). Changes in plasma GIP (**D**), blood glucose (**E**), plasma insulin (**F**), and plasma GLP1 (**G**) levels were monitored at the indicated time points. All experiments were performed with male mice after a 6-hour fast. Data are shown as the mean ± SEM (*n* = 5 or 6 mice/group). *P < 0.05, ***P < 0.001, and ****P < 0.0001, by 2-way ANOVA followed by Tukey’s post hoc analysis.

To generate mutant mice that express the GsD designer receptor selectively in enteroendocrine K cells (K-GsD mice), we crossed *ROSA26-LSL-Gs-DREADD-CRE-luc* mice (referred to herein as LSL-GsD mice) (32) with *Gip-Cre* mice that express Cre-recombinase under transcriptional control of the *Gip* promoter (33) (Figure 1A). LSL-GsD mice that lacked the *Gip-Cre* transgene served as control littermates. Since the GsD receptor carried an N-terminal hemagglutinin (HA) tag (32), we used an anti-HA antibody to detect the expression of GsD in mouse enteroendocrine cells. Immunofluorescence staining showed that GsD was only expressed by GIP-positive K cells (Figure 1B). We found that approximately 67% of GIP-positive cells expressed the GsD designer receptor (18 of a total of 27 K cells; note that K cells are very rare; see Methods for details).

To explore whether activation of GsD receptors in K cells affected endogenous GIP secretion, we carried out studies with male K-GsD mice and control littermates consuming regular chow. The 2 groups did not differ in body weight (Figure 1C). Prior to injection of DCZ, a highly selective DREADD agonist, plasma GIP levels were significantly higher in K-GsD mice than in control littermates (*P* = 0.000587, Student’s *t* test) (Figure 1D). Previous studies demonstrated that the GsD DREADD can signal, to a variable degree, in a ligand-independent fashion in certain cell types (25, 32). For this reason, the slightly elevated plasma GIP levels found in K-GsD mice in the absence of DCZ were most likely due to constitutive signaling via K cell GsD.

Strikingly, oral administration of a DCZ bolus (10 μg/kg) to K-GsD mice led to pronounced increases in plasma GIP levels 15 and 60 minutes after drug treatment (Figure 1D). DCZ administration had no significant effect on plasma GIP levels in control mice (Figure 1D). The DCZ-induced increases in plasma GIP levels in K-GsD mice did not affect blood glucose (Figure 1E) or plasma insulin (Figure 1F) levels, consistent with previous observations that GIP promotes insulin release only at elevated glucose concentrations (6, 8, 34, 35) (also see the following paragraph). Moreover, plasma GLP1 levels remained unchanged after DCZ treatment of either K-GsD mice or control littermates (Figure 1G), consistent with the selective expression of GsD in K cells.

In an additional experiment, we treated K-GsD mice and control littermates (males) orally with either glucose (2 g/kg) or DCZ (10 μg/kg) alone, or a glucose/DCZ mixture. We then monitored plasma GIP levels for up to 4 hours (Supplemental Figure 1; supplemental material available online with this article; https://doi.org/10.1172/JCI182325DS1). Treatment of control littermates with glucose alone caused a significant increase in plasma GIP levels over basal levels only at the 15-minute time point (Supplemental Figure 1A). A similar pattern was observed with glucose-treated K-GsD mice (Supplemental Figure 1A). However, because of a certain degree of ligand-independent GsD signaling (see previous paragraph), plasma GIP levels were consistently higher in glucose-treated GsD mice, as compared with glucose-treated control littermates. As expected, treatment of control littermates with DCZ alone had no significant effect on plasma GIP levels (Supplemental Figure 1B). In contrast, DCZ-treated K-GsD mice displayed higher plasma GIP levels over basal levels at the 15- and 60-minute time points. Again, because of the constitutive signaling by GsD, plasma GIP levels were higher at all time points in DCZ-treated K-GsD mice as compared with levels in DCZ-treated control littermates (Supplemental Figure 1B). The temporal pattern of GIP release observed with K-GsD mice cotreated with glucose plus DCZ was similar to that observed with DCZ-treated K-GsD mice (Supplemental Figure 1C). However, absolute plasma GIP levels were markedly higher in the cotreated K-GsD mice (note the different labeling of the *y* axis in panel 1C). Taken together, these data suggest that GsD-mediated activation of G_s_ signaling in K cells results in GIP responses that are similar in magnitude to those observed after glucose treatment. However, the GsD-mediated GIP response showed a longer duration of action. As expected, K-GsD mice cotreated with glucose plus DCZ showed more robust increases in plasma GIP levels as compared with K-GsD mice treated with either glucose or DCZ alone.

### Acute activation of K cell G_s_ signaling improves glucose tolerance in mice consuming regular chow.

**Figure 2:**
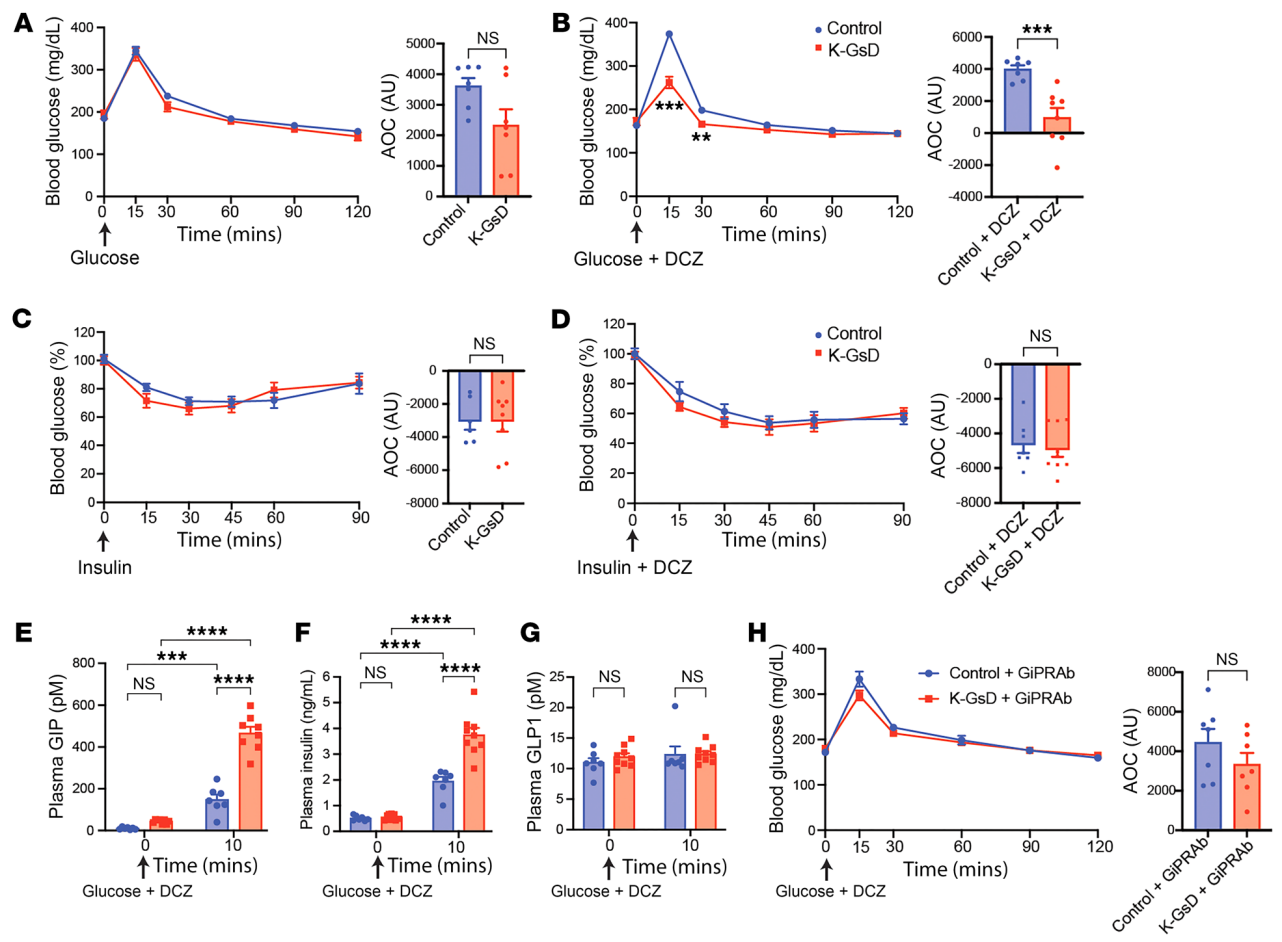
Stimulation of K cell G_s_ signaling results in improved glucose tolerance in lean K-GsD mice. (**A** and **B**) OGTTs. (**A**) K-GsD mice and control littermates received glucose only (2 g/kg). (**B**) Both groups of mice were treated with glucose plus oral DCZ (10 μg/kg). (**C** and **D**) ITTs. (**C**) K-GsD and control mice received insulin only (0.75 U/kg, i.p.). (**D**) Both groups of mice received i.p. insulin plus DCZ (10 μg/kg). Area-of-the-curve (AOC) values are given as quantitative measures of the experimental data shown in **A**–**D**. (**E**–**G**) Cotreatment of K-GsD and control mice with oral glucose plus DCZ (10 μg/kg). Plasma GIP (**E**), plasma insulin (**F**), and plasma GLP1 (**G**) levels were measured at the indicated time points. (**H**) OGTT after oral coadministration of glucose and DCZ 48 hours after treatment with a GIP receptor antibody (GIPRAb). All experiments were carried out with male mice after a 6-hour fast (the fasting period was only 4 hours for the ITT studies). Data are shown the mean ± SEM (*n* = 7 or 8 mice/group). **P < 0.01, ***P < 0.001, and ****P < 0.0001, by 2-way ANOVA followed by Tukey’s post hoc analysis (**E**–**G**) or 2-tailed, unpaired Student’s *t* test (**A**–**D** and **H**).

We next subjected K-GsD mice and control littermates (males) consuming regular chow to an oral glucose tolerance test (OGTT). Treatment of K-GsD and control mice with an oral glucose bolus alone resulted in comparable blood glucose excursions in the 2 groups of mice (Figure 2A). In contrast, following oral coadministration of glucose (2 g/kg) and DCZ (10 μg/kg), the K-GsD mice showed a striking improvement in glucose tolerance (Figure 2B), suggesting that this effect resulted from enhanced G_s_ signaling in K cells. Insulin tolerance tests (ITTs) (0.75 U/kg, i.p.) indicated that K-GsD mice and control littermates showed similar peripheral insulin sensitivity, either in the absence or presence of DCZ (Figure 2, C and D). We observed similar phenotypes when we carried out OGTTs and ITTs in female K-GsD mice and their control littermates (Supplemental Figure 2, A–D).

To explore whether the improved glucose tolerance displayed by DCZ-treated K-GsD mice (Figure 1B) was mediated by elevated GIP levels, we measured plasma GIP and insulin levels 10 minutes after oral coadministration of glucose (2 g/kg) and DCZ (10 μg/kg). This treatment resulted in significantly higher plasma GIP and insulin levels in both K-GsD mice and control littermates (Figure 2, E and F). However, these increases in plasma GIP and insulin levels were clearly more pronounced in the K-GsD mice (Figure 2, E and F). Oral coadministration of glucose and DCZ had no significant effect on plasma GLP1 levels in either of the 2 groups of mice (Figure 2G). This latter finding is not surprising, since plasma GLP1 is rapidly degraded by the actions of dipeptidyl peptidase 4 and neprilysin after oral treatment of mice with glucose (33, 36).

To provide more direct evidence that the improved glucose tolerance displayed by K-GsD mice cotreated with glucose and DCZ resulted from enhanced GIP signaling, we injected K-GsD mice and control littermates with a monoclonal, antagonistic GIP receptor antibody (Gipg013, 20 mg/kg, s.c.) (37, 38). 48 hours later, mice received an oral bolus of glucose plus DCZ, followed by monitoring of blood glucose levels. Strikingly, administration of the GIP receptor antibody completely abolished the ability of DCZ to improve glucose tolerance in K-GsD mice (Figure 2H). These data indicate that activation of G_s_ signaling in intestinal K cells improved glucose tolerance in a GIP-dependent manner.

### Acute stimulation of K cell G_s_ signaling greatly improves glucose tolerance in obese mice.

**Figure 3:**
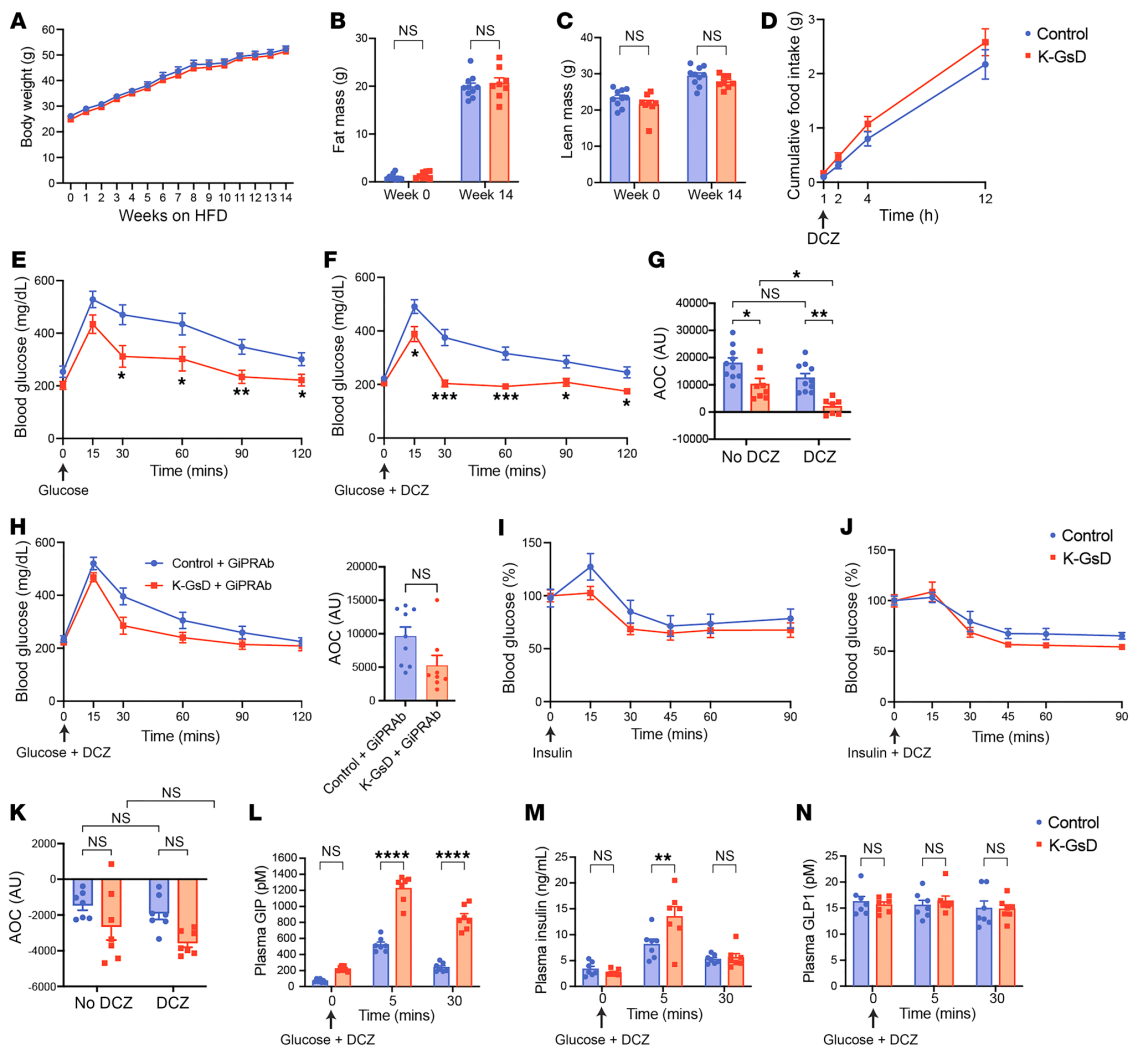
Activation of K cell G_s_ signaling greatly improves impaired glucose tolerance in obese K-GsD mice. (**A**) Body weight gain of 8-week-old K-GsD and control mice consuming a HFD for 14 weeks. (**B** and **C**) Fat mass (**B**) and lean mass (**C**) before and after HFD feeding. (**D**) Cumulative food intake measured for 12 hours after treatment with an oral bolus of DCZ (10 μg/kg in PBS). (**E**–**H**) OGTTs were administered. (**E**) Obese K-GsD mice and control littermates received glucose only (1 g/kg). (**F**) Both groups of mice were cotreated with glucose plus DCZ (10 μg/kg). (**G**) AOC values for the data shown in **E** and **F**. (**H**) Both groups of mice were cotreated with glucose plus DCZ (10 μg/kg) or saline (control), 48 hours after treatment with a GIPRAb or PBS (control). (**I**–**K**) ITTs were administered. (**I**) Obese K-GsD and control mice received insulin alone (1.5 U/kg, i.p.). (**J**) Both groups of mice received i.p. insulin plus DCZ (10 μg/kg). (**K**) AOC values for the data shown in **I** and **J**. (**L**–**N**) Cotreatment of obese K-GsD and control mice with oral glucose (1 g/kg) plus DCZ (10 μg/kg). Plasma levels of GIP (**L**), insulin (**M**), and GLP1 (**N**) were measured at the indicated time points. All experiments were performed with obese male mice after a 6-hour fast (the fasting period was only 4 hours for the ITT studies). Data are shown as the mean ± SEM (*n* = 7 – 10 mice/group). *P < 0.05, **P < 0.01, ***P < 0.001, and ****P < 0.0001, by 2-way ANOVA followed by Tukey’s post hoc analysis or 2-tailed, unpaired Student’s *t* test (**H**).

We next investigated whether acute activation of G_s_ signaling in K cells was able to improve impaired glucose tolerance in diet-induced obese mice (males). Starting at 8 weeks of age, K-GsD mice and control littermates were maintained on a HFD for 8 weeks and then subjected to a series of metabolic tests. After 14 weeks of HFD feeding, we found that the 2 groups of mice had similar body weights and body composition (lean and fat mass) (Figure 3, A–C). Moreover, food intake did not differ significantly between obese K-GsD and control mice (Figure 3D).

Interestingly, obese K-GsD mice showed a striking improvement in glucose tolerance even in the absence of DCZ (Figure 3E), most likely because the GsD receptor shows a certain degree of ligand-independent signaling (25, 32). Moreover, oral cotreatment of obese K-GsD mice with glucose (1 g/kg) plus DCZ (10 μg/kg) resulted in an even more pronounced improvement in glucose tolerance (Figure 3, F and G). To corroborate the concept that enhanced GIP signaling is responsible for the striking improvement in glucose tolerance observed with HFD K-GsD mice, we injected HFD K-GsD mice and HFD control littermates with a GIP receptor monoclonal antibody (GIPg013, 20 mg/kg, s.c.) (37, 38). Forth-eight hours later, all mice received an oral bolus of glucose plus DCZ, followed by the measurement of blood glucose levels. We found that the pronounced improvement in glucose tolerance displayed by DCZ-treated HFD K-GsD mice was markedly attenuated after administration of the GIP receptor antibody (Figure 3H). As observed with chow-fed mice, HFD K-GsD mice and control littermates showed similar decreases in blood glucose levels in i.p. ITTs, independent of the presence or absence of DCZ (Figure 3, I–K).

To test the hypothesis that the greatly improved glucose tolerance displayed by HFD K-GsD mice resulted from enhanced GIP and insulin secretion, we measured plasma GIP and insulin levels after oral coadministration of glucose (1 g/kg) and DCZ (10 μg/kg). We found that plasma levels of both GIP and insulin were significantly elevated in HFD K-GsD mice 5 minutes after cotreatment with glucose plus DCZ, as compared with HFD control littermates (Figure 3, L and M). Plasma GLP1 levels remained unaffected in both groups of mice following coadministration of oral glucose plus DCZ (Figure 3N).

Taken together, these findings strongly suggest that selective activation of G_s_ signaling in K cells stimulates the secretion of GIP and insulin when blood glucose levels are elevated, thus reversing the glucose intolerance characteristic of obese mice.

### Chronic activation of K cell G_s_ signaling improves glucose tolerance in obese mice.

**Figure 4:**
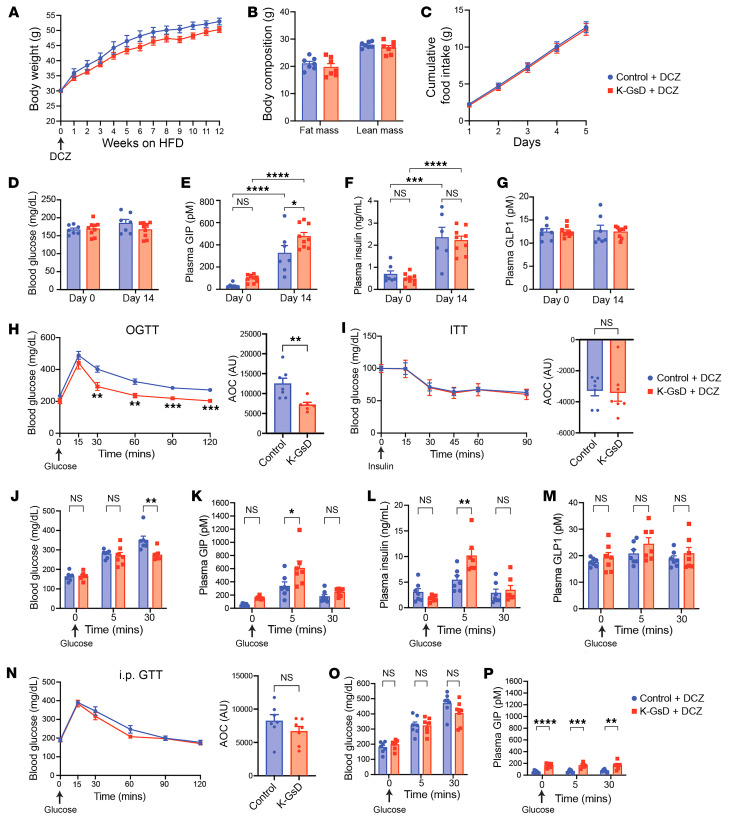
Chronic activation of G_s_ signaling in K cells improves glucose tolerance in obese K-GsD mice. (**A**) Body weight gain of 12-week-old K-GsD and control littermates maintained on a HFD and DCZ drinking water for 12 weeks. (**B**) Fat and lean mass after 11 weeks of HFD feeding. (**C**) Cumulative food intake of single-housed mice maintained on a HFD for 11 weeks. (**D**–**H**) Blood glucose (**D**) and plasma levels of GIP (**E**), insulin (**F**), and GLP1 (**G**) in nonfasted mice consuming DCZ in the drinking water. Measurements were made 14 days after the initiation of HFD feeding. (**H** and **I**) OGTT (1 g/kg) (**H**) and ITT (1.5 U/kg, i.p.) (**I**) results after 8–9 weeks of HFD feeding carried out with K-GsD and control mice consuming DCZ in the drinking water. AOC values are shown to the right in each panel. (**J**–**M**) Blood glucose (**J**) and plasma levels of GIP (**K**), insulin (**L**), and GLP1 (**M**) immediately before (time 0) and after 5 and 30 minutes of oral administration of glucose (1 g/kg) in mice consuming DCZ in the drinking water and a HFD for 10 weeks. (**N**–**Q**) Intraperitoneal GTT (1 g/kg) (**N**), blood glucose levels (**O**), and plasma levels of GIP (**P**) immediately before (time 0) and after an i.p. glucose bolus (1 g/kg) in mice consuming DCZ in the drinking water and a HFD for 11 weeks. All experiments were performed with male mice after a 6-hour fast (the fasting period was only 4 hours for the ITT studies) (**A**, **B**, and **H**–**P**). In **C**–**G** mice had free access to food. Data are shown as the mean ± SEM (*n* = 7–9 mice/group). *P < 0.05, **P < 0.01, ***P < 0.001, and ****P < 0.0001, by 2-way ANOVA followed by Tukey’s post hoc analysis (**D**–**G**, **J**–**M**, and **O**–**P**) or 2-tailed, unpaired Student’s *t* test (**A**–**C**, **H**, **I**, and **N**).

We also explored whether chronic stimulation of G_s_ signaling in K cells affects adiposity and adiposity-associated metabolic deficits in HFD K-GsD mice. To address this question, we put 12-week-old male K-GsD mice and control littermates on a HFD and added DCZ (10 mg/L) to the drinking water. We then monitored body weight gain over a 12-week period. Although body weight trended to be reduced in the K-GsD mice, this effect did not reach statistical significance (Figure 4A). Moreover, the 2 groups of mice did not differ in body composition (lean or fat mass; Figure 4B) and food intake (Figure 4C), as measured 11 weeks after initiation of HFD feeding (Figure 4C).

After 2 weeks of HFD feeding, blood glucose levels were similar in K-GsD mice and control littermates maintained on DCZ water (Figure 4D). However, plasma GIP levels were significantly elevated in both groups of mice at this point, with K-GsD mice showing markedly higher GIP levels than control mice (Figure 4E). This observation suggests that the GsD receptor–mediated GIP release was not subject to desensitization. HFD-induced increases in plasma insulin levels did not differ significantly between the 2 groups of mice (Figure 4F), consistent with previous observations that GIP-induced insulin release requires elevated blood glucose levels (34, 35). Moreover, plasma GLP1 levels were not affected in HFD K-GsD mice or control littermates under these experimental conditions (Figure 4G).

We next subjected K-GsD mice and control littermates maintained on a HFD and DCZ drinking water for 8 weeks to an OGTT. Strikingly, the K-GsD mice showed a marked improvement in glucose tolerance, as compared with the control mice (Figure 4H). Under these experimental conditions, the 2 groups of mice showed similar insulin tolerance (Figure 4I). To investigate the mechanism underlying the improved glucose tolerance displayed by the HFD K-GsD mice maintained on DCZ water, we treated both groups of mice with an oral bolus of glucose (1 g/kg), followed by the monitoring of blood glucose as well as plasma GIP, insulin, and GLP1 levels. Consistent with the OGTT data, HFD K-GsD mice showed significantly smaller increases in blood glucose levels than did the HFD control mice 30 minutes after administration of the glucose bolus (Figure 4J). Strikingly, HFD K-GsD mice showed a more robust increase in both plasma GIP and insulin levels 5 minutes after glucose treatment (Figure 4, K and L). Plasma GLP1 levels remained unaltered under these experimental conditions (Figure 4M). The time course of the observed changes in blood glucose and plasma GIP and insulin levels strongly suggests that GsD-mediated increases in GIP release trigger enhanced insulin secretion, which in turn led to reduced glucose-induced hyperglycemic responses.

While HFD K-GsD mice consuming DCZ water showed greatly improved glucose tolerance in the OGTT (Figure 4H), this effect was not observed when HFD K-GsD mice and control littermates were subjected to an intraperitoneal GTT (Figure 4, N and O). In agreement with this observation, an i.p. glucose bolus (1 g/kg) did not lead to a rise in plasma GIP levels in both groups of mice (Figure 4P). However, given the ligand-independent activity of the GsD receptor (25, 32), HFD K-GsD mice showed elevated plasma GIP levels at all time points (including time 0) as compared with their HFD-fed control littermates (Figure 4P).

Since GIP has been implicated in the regulation of energy homeostasis (reviewed in ref. 39), we performed a series of indirect calorimetry studies at thermoneutrality (30°C) and ambient temperature (22°C). We found that HFD K-GsD mice consuming DCZ water did not differ from their control littermates in total energy expenditure, respiratory exchange ratio (RER), and total ambulatory activity at 30°C or 22°C (Supplemental Figure 3). In sum, these data indicate that chronic activation of G_s_ signaling in K cells leads to enhanced circulating GIP levels that promote euglycemia but have no effect on body weight, food intake, or energy expenditure.

### Chronic activation of K cell G_s_ signaling greatly reduces hyperglycemia in a mouse model of diabetes.

We next examined whether chronic activation of K cell G_s_ signaling might improve glucose homeostasis in a mouse model of diabetes. Specifically, we treated K-GsD mice and control littermates (8-week-old males) with a relatively low dose of streptozotocin (STZ) for 5 consecutive days (50 mg/kg i.p. daily) (40, 41). Previous studies demonstrated that this treatment protocol does not destroy all β cells but reduces β cell mass by approximately 80% (40, 41), thus mimicking the pronounced decrease in β cell mass characteristic in advanced T2D (42). In agreement with this observation, a recent study demonstrated that β cells from mice treated with multiple low doses of STZ show changes in gene expression similar to those seen in β cells in human T2D (43).

**Figure 5:**
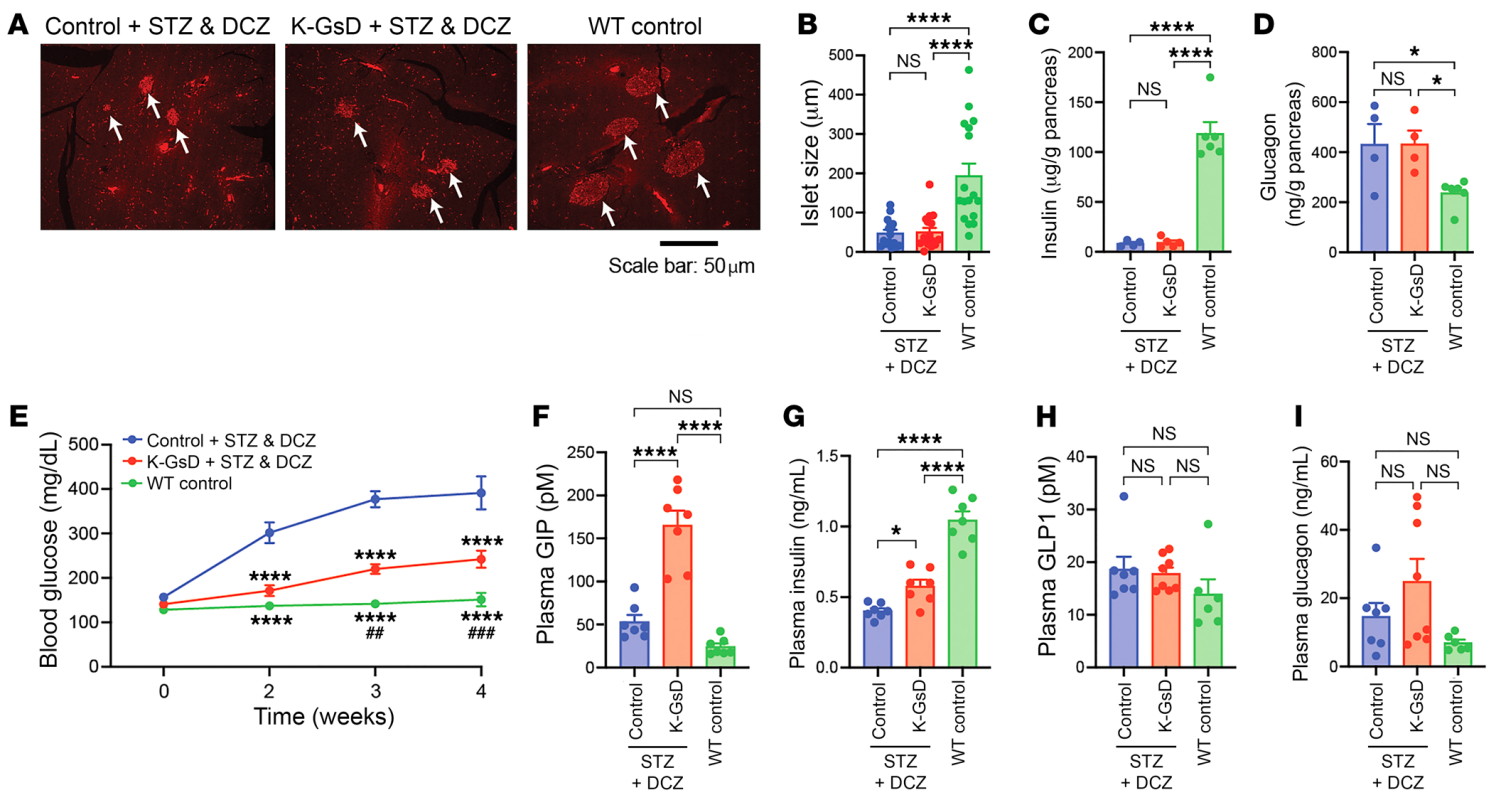
Chronic activation of K cell G_s_ signaling greatly reduces STZ-induced hyperglycemia. (**A**) Representative immunofluorescence images showing insulin staining of pancreatic slices from mice of the indicated phenotypes. White arrows point to pancreatic islets. Healthy, non-STZ-treated WT mice were included for control purposes. Scale bar: 50 μm. (**B**) Quantification of islet size at the end of the treatment period. (**C** and **D**) Pancreatic content of insulin (**C**) and glucagon (**D**) at the end of the treatment period. (**E**) Suppression of STZ-induced hyperglycemia by cotreatment of K-GsD mice with STZ and DCZ water (10 mg/L). (**F**–**I**) Plasma levels of GIP (**F**), insulin (**G**), GLP1 (**H**), and glucagon (**I**) at the end of the treatment period. Blood glucose and plasma hormones were measured after a 5-hour fast. In **B**, at least 15 islets from 3 different mice per group were analyzed (*n* = 4–6 mice/group for **C** and **D**, and 6–8 mice per group for **E**–**I**, respectively). Data are shown as the mean ± SEM. *P < 0.05 and ****P < 0.0001, by 1-way ANOVA (**B**–**D** and **F**–**I**) and 2-way ANOVA (**E**) followed by Tukey’s post hoc analysis, respectively. (**E**) ****P < 0.0001 relative to control + STZ&DCZ, and ^##^*P* < 0.01 and ^###^*P* < 0.001 relative to K-GsD STZ&DCZ, respectively.

All mice received DCZ via the drinking water (10 mg/L), starting on the first day of STZ treatment, except for WT control mice, which received no treatment at all. Four weeks after the first STZ injection, both K-GsD mice and control littermates showed a reduction of approximately 75% in islet size as compared with WT mice treated with neither STZ nor DCZ (Figure 5, A and B). Moreover, at this time point, pancreatic insulin and glucagon content did not differ significantly between K-GsD mice and control littermates treated with STZ plus DCZ (STZ+DCZ) (Figure 5, C and D). However, in comparison with WT mice that had not been treated with STZ+DCZ, pancreatic insulin content was significantly reduced, whereas pancreatic glucagon content was significantly increased (Figure 5, C and D). Strikingly, although STZ+DCZ-treated control mice developed severe hyperglycemia during the 4-week observation period, this response was greatly reduced in STZ+DCZ-treated K-GsD mice (Figure 5E). The different groups of mice showed similar body weights throughout the 4-week observation period (Supplemental Figure 4).

Interestingly, STZ+DCZ-treated K-GsD mice showed significantly increased plasma GIP and insulin levels 4 weeks after the start of the experiment (Figure 5, F and G), as compared with the STZ+DCZ-treated control littermates. Plasma levels of GLP1 and glucagon (Figure 5,H and I) did not differ significantly between the different groups of mice. Taken together, these data indicate that chronic stimulation of K cell G_s_ signaling potently counteracted the development of hyperglycemia in a mouse model of diabetes, most likely via GIP-mediated stimulation of insulin secretion.

We next explored the possibility that treatment of WT mice with mouse [D-Ala^2^]GIP (41, 44), a relatively stable GIP analog (45, 46), could reduce STZ-induced hyperglycemia in a fashion similar to that observed with STZ+DCZ-treated K-GsD mice. In agreement with the outcome of a recent study (47), we found that i.p. treatment of WT mice for 4 weeks with [D-Ala^2^]GIP (24 nmoles/kg per dose; 2 injections per day; injection times: 9 am and 6 pm) had no significant effect on STZ-induced hyperglycemia (Supplemental Figure 5). Since previous studies have shown that endogenous GIP is rapidly metabolized to GIP (3–42), which can act as a weak partial agonist at GIP receptors (48), it is possible that GIP (3–42) contributes to the beneficial metabolic effects observed after G_s_-mediated stimulation of GIP release in this STZ diabetes model.

### Generation and metabolic characterization of K-Gs–KO mice.

**Figure 6:**
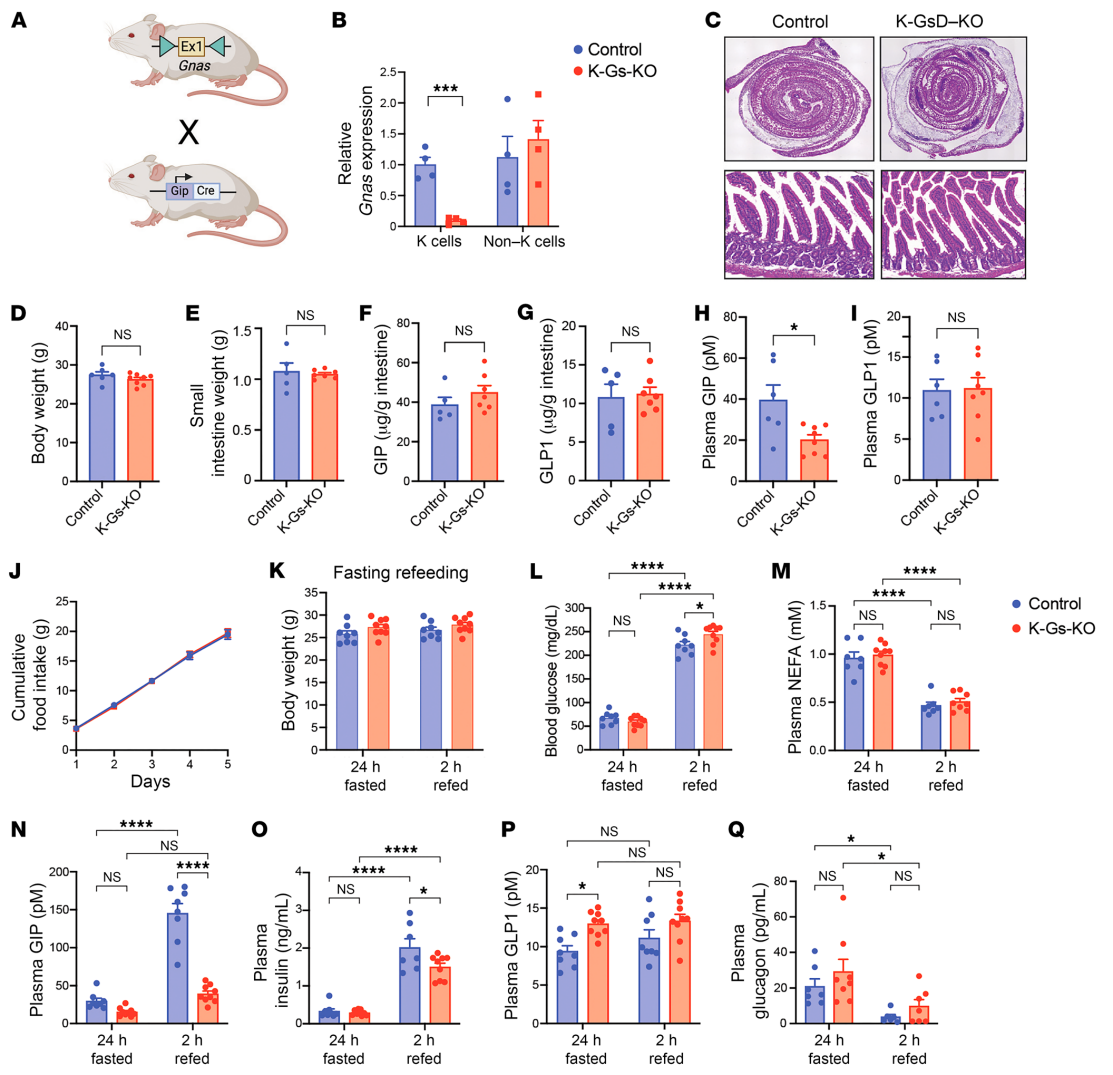
Metabolic studies with K-Gs–KO mice maintained on regular chow. (**A**) Schematic depicting the generation of K-Gs–KO mice that lack Gα_s_ selectively in enteroendocrine K cells. (**B**) Transcript levels of *Gnas*, the gene that encodes Gα_s_, measured with RNA prepared from duodenal FACS-sorted K cells and non–K cells. (**C**) Representative images of H&E staining experiments showing that the lack of Gα_s_ in K cells did not affect the overall morphology of the proximal intestine/duodenum. Original magnification, ×40 (top row) and ×60 (bottom row). (**D**–**G**) K-Gs–KO mice and control littermates showed similar body weights (**D**), whole intestine weights (**E**), and intestinal contents of GIP (**F**) and GLP1 (**G**). (**H** and **I**) K-Gs–KO mice showed reduced plasma GIP levels (**H**), but unchanged plasma GLP1 levels (**I**). (**J**) Cumulative food intake measured over 5 days in single-housed mice. (**K**–**Q**) Metabolic parameters of K-Gs–KO mice and control littermates after refeeding following a 24-hour fast. Body weights (**K**), blood glucose levels (**L**), and plasma levels of NEFAs (**M**), GIP (**N**), insulin (**O**), GLP1 (**P**), and glucagon (**Q**). All experiments were performed with male mice. In **B**–**I**, mice were subjected to a 6-hour fast. Data are shown as the mean ± SEM (*n* = 7–9 mice/group). *P < 0.05, ***P < 0.001, and ****P < 0.0001, by 2-way ANOVA followed by Tukey’s post hoc analysis (**K**–**Q**) or 2-tailed, unpaired Student’s *t* test (**B** and **D**–**J**).

We next investigated whether the lack of K cell G_s_ signaling resulted in metabolic phenotypes that were opposite to those displayed by DCZ-treated K-GsD mice. To address this question, we generated mice that selectively lacked Gα_s_ in intestinal K cells (K-Gs–KO mice). To obtain this mouse strain, we crossed *Gnas^fl/fl^* mice (49) with *Gip-Cre* mice (33) (Figure 6A). The resulting *Gnas^fl/+^* *Gip-Cre* mice were then backcrossed with *Gnas^fl/fl^* mice to generate *Gnas^fl/fl^* *Gip-Cre* mice (K-Gs–KO) and control littermates (*Gnas^fl/fl^*). To study the efficiency of Cre-mediated inactivation of *Gnas*, we followed a previously published FACS protocol (50) to obtain K cells from the mouse duodenum. As expected, duodenal K cells prepared from K-Gs–KO mice showed an approximately 90% reduction in *Gnas* expression, as compared with non–K cells present in the mouse duodenum (Figure 6B).

H&E staining studies showed that the lack of K cell G_s_ had no obvious effect on the overall morphology of the proximal intestine (duodenum, Figure 6C). Moreover, K-Gs–KO mice and control littermates did not differ in body weight (Figure 6D), small intestine weight (Figure 6E), or intestinal content of GIP and GLP1 (Figure 6, F and G). However, plasma GIP levels were significantly reduced (by ~50%) in the K-Gs–KO mice (Figure 6H). Plasma GLP1 levels did not differ significantly between K-Gs–KO mice and control littermates (Figure 6I). Food intake of singly housed mice remained unaffected by the lack of K cell Gα_s_ signaling (Figure 6J).

We next subjected K-Gs–KO mice and control littermates to a 24-hour fast, followed by a 2-hour refeeding period (diet: regular chow). The 2 groups of mice showed no significant differences in body weight under these experimental conditions (Figure 6K). At the end of the 2-hour refeeding period, the K-Gs–KO mice showed a modest but significant increase in blood glucose levels as compared with their control littermates (Figure 6L). The plasma levels of nonesterified fatty acids (NEFAs) did not differ between the 2 groups of mice, both in the fasted state and after the refeeding period (Figure 6M).

In control mice, plasma GIP levels were greatly increased at the end for the 2-hour refeeding period (Figure 6N). In striking contrast, plasma GIP levels were not elevated in K-Gs–KO mice under these experimental conditions (Figure 6N), suggesting that the lack of G_s_-stimulated GIP release was responsible for the greater increase in blood glucose levels observed with the K-Gs–KO mice after the refeeding period. In agreement with this notion, the increase in plasma insulin levels triggered by refeeding was significantly less pronounced in K-Gs–KO mice as compared with their control littermates (Figure 6O). In the fasted state, plasma GLP1 levels were significantly increased in the absence of K cell G_s_ signaling (Figure 6P). However, plasma GLP1 levels did not differ significantly between K-Gs–KO mice and control littermates after the 2-hour refeeding period (Figure 6P) (36). Plasma glucagon levels did not differ between the two groups of mice, either in the fasting state or after the refeeding period (Figure 6Q).

### Inhibition of G_s_ signaling in K cells reduces GIP secretion but does not affect glucose homeostasis in lean and diet-induced obese mice.

**Figure 7:**
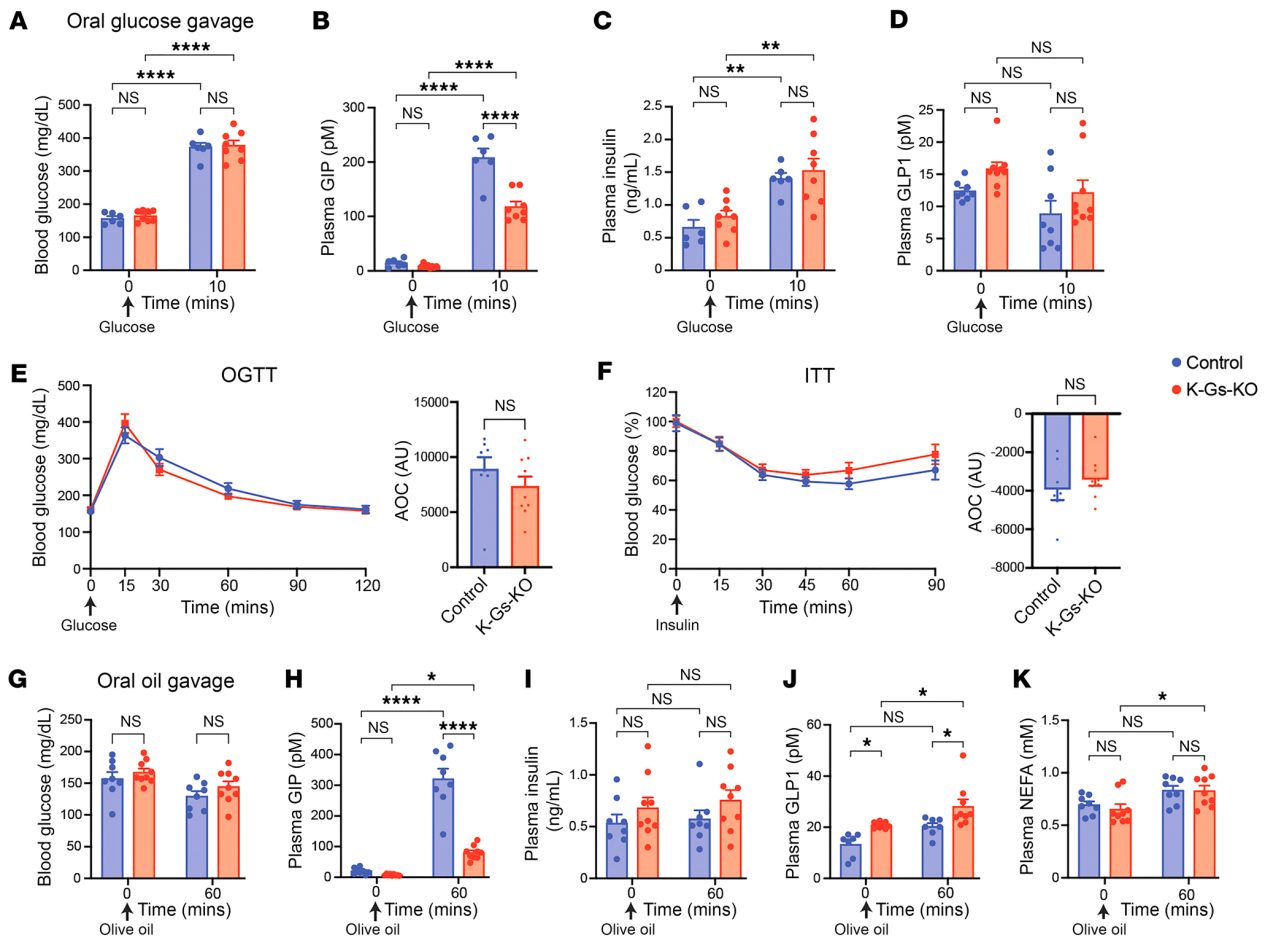
Reduced plasma GIP levels in K-Gs–KO mice do not affect whole-body glucose homeostasis. All studies were carried out with male K-Gs–KO mice and control littermates consuming regular chow. (**A**–**D**) Blood glucose levels (**A**) and plasma levels of GIP (**B**), insulin (**C**), and GLP1 (**D**), immediately before (time 0) and 10 minutes after treatment with an oral glucose bolus (2 g/kg). (**E** and **F**) OGTTs (2 g/kg) (**E**) and ITTs (0.75 U/kg, i.p.) (**F**) were administered. AOC values are shown to the right in each panel. (**G**–**K**) Blood glucose levels (**G**) and plasma levels of GIP (**H**), insulin (**I**), GLP1 (**J**), and NEFAs (**K**), immediately before (time 0) and 1 hour after oral gavage with olive oil (10 μL/g). All experiments were performed with male mice after a 6-hour fast (the fasting period was only 4 hours for the ITT studies). Data are shown as the mean ± SEM (*n* = 7–10 mice/group). *P < 0.05, **P < 0.01, and ****P < 0.000, by 2-way ANOVA followed by Tukey’s post hoc analysis (**A**–**D** and **G**–**K**) or 2-tailed, unpaired Student’s *t* test (**E** and **F**).

To study the effects of inactivating G_s_ signaling in K cells on systemic glucose homeostasis, K-Gs–KO mice and control littermates consuming regular chow received an oral glucose bolus (2 g/kg). Ten minutes later, blood glucose levels were elevated to a similar degree in both groups of mice (Figure 7A). However, glucose-induced elevations in plasma GIP levels were reduced by approximately 50% in the K-Gs–KO mice (Figure 7B). Glucose-dependent increases in plasma insulin levels were comparable in magnitude between the 2 groups of mice (Figure 7C). Plasma GLP1 levels remained unchanged under these experimental conditions (Figure 7D).

Similarly, K-Gs–KO mice and control littermates maintained on regular chow showed similar blood glucose excursions in an OGTT (Figure 7E). Likewise, exogenously administered insulin (0.75 U/kg, i.p.) caused similar decreases in blood glucose levels in the 2 groups of mice (ITT, Figure 7F). We obtained similar results with female K-Gs–KO and control mice (reduced plasma GIP but unaltered plasma GLP1 and blood glucose excursions in OGTT and ITT assays) (Supplemental Figure 6, A–D).

Since the ingestion of triglycerides strongly stimulates the release of GIP from K cells (6, 51), we next treated K-Gs–KO mice and control littermates with an oral bolus of olive oil (10 μL/g), followed by the measurement of blood glucose and plasma hormone and metabolite levels 60 minutes later. At this time point, blood glucose levels did not differ significantly between the 2 groups of mice (Figure 7G). In control mice, olive oil administration resulted in a very robust increase in plasma GIP levels (Figure 7H). Strikingly, this effect was greatly reduced in K-Gs–KO mice (Figure 7H). Treatment with olive oil had no significant effect on plasma insulin levels in either group of mice under these experimental conditions (Figure 7I). However, in K-Gs–KO mice, olive oil administration caused a small but significant increase in plasma GLP1 levels (Figure 7J), perhaps resulting from the activation of a compensatory pathway caused by low circulating GIP levels (Figure 7H). Plasma levels of NEFAs were similar between the 2 two groups of mice prior to and 60 minutes after olive oil administration (Figure 7K).

To investigate whether the lack of K cell G_s_ signaling affected adiposity and adiposity-linked metabolic deficits in mice consuming a HFD, we subjected K-Gs–KO mice and control littermates that had been maintained on a HFD for 14 weeks to a series of metabolic tests. Under these experimental conditions, the 2 groups of mice did not differ significantly in body weight gain, body composition (lean versus fat mass), or daily food intake (Supplemental Figure 6, E–H). HFD-fed K-Gs–KO mice and control littermates also showed comparable blood glucose excursions in the OGTTs and ITTs (Supplemental Figure 6, I and J). Moreover, following, an oral glucose bolus (1 g/kg), blood glucose (Supplemental Figure 6K) and plasma insulin levels (Supplemental Figure 6M) were elevated to a similar extent in the 2 groups of mice. However, glucose-stimulated GIP secretion was strongly reduced in the HFD-fed K-Gs–KO mice 10 minutes after treatment with the oral glucose bolus (Supplemental Figure 6L), as observed in K-Gs–KO mice consuming regular chow (Figure 7B). Plasma GLP1 levels remained unchanged in the 2 groups under these experimental conditions (Supplemental Figure 6N).

Since the HFD-fed K-Gs–KO mice did not display any detectable deficits in glucose homeostasis under different experimental conditions, we speculated that signaling via GLP1, the second major incretin, was able to compensate for impaired GIP release caused by the lack of K cell G_s_ signaling. To test this hypothesis, we treated HFD-fed K-Gs–KO and control littermates with an antagonistic GLP1 receptor antibody (Glp10017, 19.2 mg/kg, s.c.) (37) or vehicle (PBS), followed by an OGTT. We found that administration of the GLP1 receptor antibody led to strikingly enhanced blood glucose excursions in both K-Gs–KO and control mice (Supplemental Figure 6O). However, the magnitude of this effect was similar in the 2 groups of mice in comparison with the PBS-treated mice. These observations suggest that the relatively low plasma GIP levels characteristic of K-Gs–KO mice under different experimental conditions suffice to maintain euglycemia. Taken together, these data indicate that inhibition of K cell G_s_ signaling reduced GIP secretion but did not affect glucose tolerance in lean or obese mice.

### The lack of K cell G_s_ signaling does not affect the ability of a GPR40 agonist to stimulate GIP secretion.

A recent study demonstrated that agonist activation of a G_q_-coupled DREADD selectively expressed in mouse K cells leads to a pronounced increase in GIP secretion (52). Previous work has also shown that GPR40 (alternative name: FFAR1), a G_q_-coupled receptor that is activated by long-chain fatty acids, is enriched in enteroendocrine K cells (reviewed in ref. 6). To test the possibility that FFAR1 activity can promote GIP release in K-Gs–KO mice, we treated K-Gs–KO mice and control littermates with AM1638, a highly selective and efficacious FFAR1 agonist (53, 54). We found that oral AM1638 treatment (30 mg/kg) (53) of K-Gs–KO mice resulted in a robust increase in GIP secretion (Supplemental Figure 7). The magnitude of this response was not significantly different from that observed in control littermates (Supplemental Figure 7). Taken together these data suggest that receptor-activated G_q_ signaling in K cells remains as a major stimulant of GIP secretion in K-Gs–KO mice.

### G_s_-coupled receptors endogenously expressed by K cells.

To explore which endogenous G_s_-coupled receptors are expressed by mouse and human K cells, we mined previously published scRNA-Seq data (55, 56). The 10 mouse and human G_s_-coupled receptors that showed the highest expression levels are displayed in Supplemental Figure 8. In mouse K cells, *Gpr119* was by far the most abundant G_s_-coupled receptor (as far as gene expression levels are concerned). On the other hand, in human K cells, *GPBAR1* was the predominant G_s_-coupled receptor transcript. Among the receptors listed in Supplemental Figure 8, five G_s_-coupled receptors are expressed in both mouse and human K cells including the G protein–coupled bile acid receptor 1 (*GPBAR1*), the prostanoid EP4 receptor (*PTGER4*), the VPAC_1_ receptor (*VIPR1*), the GIP receptor (*GIPR*), and the A_2A_ adenosine receptor (*ADORA2A*). The G_s_-coupled receptors expressed by human K cells can be considered potential targets for drugs able to stimulate GIP secretion for therapeutic purposes.

## Discussion

During the past decade, highly potent and efficacious GLP1/GIP receptor co-agonists (e.g., tirzepatide) (57–59) have been developed for the treatment of T2D and obesity (60–62). Interestingly, a recent study demonstrated that the ability of tirzepatide to promote insulin release from human islets requires the presence of islet GIP receptors (63). These and other studies have generated renewed strong interest in exploring the physiological and pathophysiological roles of GIP. In the present study, we tested the hypothesis that selective stimulation of GIP release from enteroendocrine K cells might be able to restore euglycemia in obese mice and in a mouse model of T2D (STZ-induced reduction in β cell mass).

The GIP-producing K cells, like other enteroendocrine cells, express dozens of GPCRs that are predicted to modulate GIP release (6, 56, 64). GPCRs represent very attractive therapeutic targets, primarily due to their cell-surface localization and their ability to modulate the activity of many important cellular signaling pathways (65). Since the individual members of the GPCR superfamily are usually expressed by multiple tissues and cell types (23), it has not been possible to stimulate a specific GPCR or G protein signaling pathway in K cells in vivo by using classical pharmacological techniques.

To overcome this obstacle, we decided to apply a chemogenetic approach involving the use of DREADD technology (26, 27, 29). Miedzybrodzka et al. (66) recently showed that treatment of human duodenal organoids with forskolin, a potent activator of adenylyl cyclase, was able to strongly stimulate the release of GIP. Prompted by this observation, we decided to test the hypothesis that selective activation of G_s_ signaling (active Gα_s_ is a potent stimulant of adenylyl cyclase) leads to enhanced GIP release in vivo. Specifically, we generated a mouse model that expressed a GsD (25) selectively in K cells (K-GsD mice) (Figure 1, A and B).

In agreement with our hypothesis, acute treatment of either lean or obese K-GsD mice with DCZ, a highly selective DREADD agonist (34), led to marked increases in plasma GIP levels (Figure 1D, Figure 2E, and Figure 3L). In the absence of coadministered glucose, these elevated GIP levels had no effect on insulin secretion or glucose homeostasis in general. This observation is consistent with previous findings that GIP triggers insulin secretion only when blood glucose levels are elevated (6, 8, 34, 35). In contrast, oral cotreatment of lean or obese K-GsD mice with DCZ plus glucose resulted in striking increases in plasma GIP and insulin levels and greatly improved glucose tolerance. Since the observed increases in plasma GIP and insulin levels preceded the beneficial effect on blood glucose levels (Figure 2, E and F, Figure 3, L and M, and Figure 4, K and L), our data strongly support the concept that activation of K cell G_s_ signaling stimulates the secretion of GIP, which then acts on β cell GIP receptors to promote the release of insulin, followed by a reduction of blood glucose levels and improved glucose homeostasis. The observation that stimulation of K cell G_s_ signaling was able to restore euglycemia in obese, glucose-intolerant mice is of particular translational interest (Figure 3F).

In general, obesity is associated with marked increases in plasma GIP levels (12, 67, 68) (also see Figure 4E). Because of this finding, it is widely assumed that elevated GIP levels are linked to GIP receptor desensitization (reviewed in refs. 12, 69). However, our data clearly show that further increasing plasma GIP levels in obese mice leads to greatly improved glucose homeostasis (Figure 3F and Figure 4H), suggesting that GIP receptors remain responsive in the obese state despite high circulating plasma GIP levels.

Early work showed that administration of exogenous GIP failed to stimulate insulin secretion in patients with T2D (70). This finding prompted the speculation that β cell GIP receptors are desensitized or downregulated in T2D due to high circulating levels of GIP (reviewed in refs. 12, 69). However, Stensen et al. (21) recently showed that treatment of diabetic individuals with a selective GIP receptor antagonist lowered plasma insulin levels after a mixed meal, indicating that endogenous GIP retains considerable insulinotropic activity in T2D. In agreement with this finding, we demonstrated that chronic DCZ treatment of obese K-GsD mice with impaired glucose homeostasis resulted in increases in plasma levels of both GIP and insulin (Figure 4, E and F). These hormonal changes were accompanied by a marked improvement in glucose tolerance, strongly suggesting that GIP receptor–mediated insulin release is not subject to desensitization under these diabetogenic conditions and that chronic activation of K cell G_s_ signaling may prove useful to restore euglycemia in T2D.

We also investigated whether chronic activation of K cell G_s_ signaling might improve glucose homeostasis in a mouse model of T2D (STZ-induced reduction in β cell mass). Strikingly, while STZ+DCZ-treated control mice developed severe hyperglycemia, this response was greatly reduced in STZ+DCZ-treated K-GsD mice (Figure 5E). STZ+DCZ-treated K-GsD mice showed significantly elevated plasma GIP and insulin levels, as compared with STZ+DCZ treated control mice (Figure 5, F and G). These data suggest that the beneficial metabolic effects observed after chronic activation of K cell G_s_ signaling in this mouse model of diabetes were mediated by GIP-dependent stimulation of insulin secretion.

Many studies have shown that a fat-rich meal strongly stimulates the secretion of GIP from K cells (reviewed in refs. 6, 51, 64). In agreement with this finding, we showed that oral administration of olive oil resulted in increased plasma GIP levels in control mice (Figure 7H). Interestingly, this response was greatly attenuated in K-Gs–KO mice (Figure 7H), suggesting that long-chain fatty acids or monoacylglycerols released by the enzymatic breakdown of the triglycerides contained in olive oil primarily act by stimulating K cell G_s_ signaling to exert their stimulatory effect on GIP release. K cells express GPCRs for several long-chain fatty acids and monoacylglycerols, including FFAR1 (GPR40), FFAR4 (GR120), and GPR119 (64). Whereas the activation of FFAR1 and FFAR4 by long-chain fatty acids preferentially leads to the stimulation of G proteins of the G_q_ family, the binding of monoacylglycerols to GPR119 triggers the activation of G_s_ (64, 71). Taken together, these data suggest that the ingestion of a fat-rich meal triggers GIP release primarily by promoting K cell G_s_ signaling, probably involving the activation of GPR119. In future studies, we are planning to further test this hypothesis by generating and analyzing K cell–specific *Gpr119*-KO mice.

In agreement with the phenotypes displayed by the K-GsD mice, mice selectively lacking Gα_s_ in K cells (K-Gs–KO mice) showed marked reductions in plasma GIP levels, consistent with the important role of G_s_ in regulating K cell function. Refeeding studies showed that control mice had markedly elevated plasma GIP levels at the end of the 2-hour refeeding period, whereas plasma GIP levels remained unchanged in K-Gs–KO mice under these experimental conditions (Figure 6N). In agreement with this observation, the refeeding-induced hyperglycemia was significantly elevated in K-Gs–KO mice as compared with their control littermates (Figure 6L). This observation clearly indicates that K cell G_s_ signaling plays a physiological role in suppressing meal-induced blood glucose excursions. However, K-Gs–KO mice did not show significant changes in body weight, food intake, glucose tolerance, or insulin sensitivity, suggesting that a reduction of plasma GIP levels by approximately 50% does not affect other metabolic parameters, at least not in mice.

As mentioned in the previous paragraph, K-Gs–KO mice showed residual GIP production. It is well documented that GIP release is triggered not only by receptor-mediated activation of G_s_ signaling in K cells, but also by activation of receptors linked to G proteins of the G_q_ family, including FFAR1 (Supplemental Figure 7) (reviewed in ref. 6). Moreover, many nutrients including glucose, peptides, and amino acids promote GIP secretion via G protein–independent mechanisms (6), explaining why GIP release still occurs in K-Gs–KO mice. The observation that reduced GIP secretion did not affect glucose tolerance in lean female and obese male K-Gs–KO mice is most likely due to the ability of GLP1 and/or other signaling molecules to compensate for the reduction in plasma GIP levels caused by the lack of K cell G_s_ signaling.

While most studies described in this manuscript were performed with male mice, several key experiments were repeated with female mice. These studies showed that DCZ-treated female K-GsD and K-Gs–KO mice had metabolic changes similar to those seen in their male counterparts (Supplemental Figure 2, A–D, and Supplemental Figure 6, A–D).

Interestingly, the analysis of published scRNA-Seq data showed that both mouse and human K cells express many GPCRs that preferentially couple to G_s_. Mouse K cells express particularly high levels of *Gpr119*, which codes for a G_s_-coupled receptor that is activated predominantly by breakdown products of triglycerides (Supplemental Figure 8). In the present study, we demonstrated that olive oil–induced GIP release was greatly reduced in K-Gs–KO mice (Figure 7H). A similar phenotype was observed when olive oil was given to *Gpr119*-KO mice (72). Taken together, these findings suggest that triglyceride-induced GIP release was primarily mediated by the activation of K cell Gpr119, at least in the mouse.

We also identified 5 G_s-_coupled receptors that are expressed in both mouse and human K cells (Supplemental Figure 8). The G_s-_coupled receptors expressed by human K cells, including the *GPBAR1* which was expressed at particularly high levels, could emerge as useful targets for the development of drugs aimed at enhancing endogenous GIP release for therapeutic purposes.

Of note, both mouse and human K cells also expressed G_s_-coupled GIP receptors (Supplemental Figure 8). In the present study, we demonstrated that stimulation of G_s_ signaling in K cells resulted in a pronounced increase in GIP release. GIP secreted from K cells is predicted to activate K cell GIP receptors, leading to a further enhancement of GIP release in a positive feedback loop.

In conclusion, metabolic studies with K-GsD- and K-Gs–KO mice have led to several important findings regarding the mechanisms of GIP release and the metabolic benefits of stimulating endogenous GIP release. Clearly, these data provide a rational basis for the development of drugs that can promote K cell G_s_ signaling for the treatment of T2D and related metabolic disorders.

## Methods

### Sex as a biological variable.

Our study examined male and female mice, and similar findings were obtained for both sexes.

### Mouse maintenance.

Mice were group-housed at 23°C and fed ad libitum on a 12-hour light/12-hour dark cycle. Most studies were carried out with mice consuming a regular chow diet (7022 NIH-07, 15% kcal fat, energy density 3.1 kcal/g; Envigo). A subgroup of mice was maintained on a high-fat diet (HFD) (F3282, 60% kcal from fat, energy density, 5.5 kcal/g; Bioserv) after reaching 8 weeks of age. Metabolic studies were performed with mice that were at least 8 weeks old.

### Generation of K cell–specific Gs DREADD (GsD) and Gα_s_-KO mice.

To generate K cell–specific GsD mice, homozygous *ROSA26-LSL-Gs-DREADD-CRE-luc* (referred to herein as LSL-GsD) mice (32) were crossed with *Gip-Cre* mice that expressed Cre recombinase under transcriptional control of the *Gip* promoter (33). *Cre*-positive hemizygous LSL-GsD mice (K-GsD mice) are predicted to express the GsD designer receptor selectively in K cells.

LSL-GsD mice lacking the *Cre* transgene served as control littermates.

To inactivate *Gnas* (encoded protein: Gα_s_) selectively in K cells, we crossed ﬂoxed *Gnas* mice (*Gnas^ﬂ/ﬂ^* mice) (49) with *Gip-Cre* mice (33). The resulting *Cre*-positive *Gnas^ﬂ/+^* mice were then backcrossed with *Gnas^ﬂ/ﬂ^* mice. *Cre*-positive *Gnas^ﬂ/ﬂ^* mice (K-Gs–KO mice) are predicted to harbor an inactive version of *Gnas* selectively in K cells. *Gnas^ﬂ/ﬂ^* mice that did not carry the *Cre* transgene were used as control littermates. Mouse tail DNA was used for PCR genotyping of *Gip-Cre*, *Gnas^ﬂ/ﬂ^*, and LSL-GsD mice. PCR reactions were carried out using standard procedures. Primer sequences are listed in Supplemental Table 1.

All mice used for these matings had been backcrossed for at least 7 times onto a C57BL/6 background.

All other experimental procedures are described in the Supplemental Methods.

### Statistics.

Data are expressed as the mean ± SEM for the indicated number of observations. Data were assessed for statistical significance by 2-way-ANOVA, followed by the indicated post hoc tests, or by 2-tailed, unpaired Student’s *t* test, as appropriate. A *P* value of less than 0.05 was considered statistically significant. The specific statistical tests that were used are indicated in the figure legends.

### Study approval.

All animal experiments were conducted according to the NIH Guidelines for Animal Research and were approved by the IACUC of the NIDDK, NIH.

### Data availability.

All data are available in the main text or the supplemental materials. Values for all data points in graphs are reported in the Supporting Data Values file.

## Author contributions

ABO and JW conceived and designed the study. ABO performed most of the experiments, with help from LL, YC, HL, NG, OG, and JEL. FR, FMG, MC, LSW, and JEC provided mouse models or reagents and gave helpful advice throughout this study. ABO wrote the first draft of the manuscript. ABO and JW jointly finalized the manuscript.

## Supplementary Material

Supplemental data

Supporting data values

## Version 1 10/22/2024

In-Press Preview

## Version 2 12/16/2024

Electronic publication
