# Supplementary material for: Activation of Gs signaling in mouse enteroendocrine K cells greatly improves obesity- and diabetes-related metabolic deficits
Source: J Clin Invest. 2024 Oct 22;134(24):e182325. doi: 10.1172/JCI182325 (PMC11649064; doi:10.1172/JCI182325)
Supplement: Supplemental data [file jci-134-182325-s008.pdf]

Supplemental Data

Activation of G_s signaling in mouse enteroendocrine K-cells greatly improves obesity- and diabetes-related metabolic deficits

Antwi-Boasiako Oteng, Liu Liu, Yinghong Cui, Oksana Gavrilova, Huiyan Lu, Min Chen, Lee S. Weinstein, Jonathan E. Campbell, Jo E. Lewis, Fiona M. Gribble, Frank Reimann, and Jürgen Wess

Supplemental Methods

Physiological studies.

Body composition (lean and fat mass) was determined via EchoMRI (EchoMRI100H; EchoMRI LLC). In acute DCZ challenge tests, K-GsD mice and control littermates received a single dose of DCZ (10 μg/kg in PBS orally or via i.p. injection). Prior to DCZ treatment, mice had free access to food or had been fasted for 6 hours. For chronic DCZ administration studies, DCZ was added to the drinking water at a concentration of 10 mg/l.

Oral glucose tolerance and intraperitoneal (i.p.) glucose tolerance tests (OGTT and ipGTT, respectively) were conducted after a 6-hour fast. Mice were treated with a glucose bolus (2 g/kg for chow-fed mice; 1 g/kg for HFD mice), either in the presence or absence of oral DCZ (10 μg/kg). Blood glucose levels were measured immediately prior to glucose administration and at defined post-treatment time points. To study glucose-stimulated insulin secretion, mice were treated with glucose via oral gavage (2 g/kg for chow-fed mice; 1 g/kg for HFD mice), either in the absence or presence of oral DCZ (10 μg/kg). Blood was collected immediately before and at defined post-treatment time points for the measurement of plasma incretin, insulin, and glucagon levels.

For insulin tolerance tests (ITT), K-GsD mice and control littermates that had been fasted for 4 hours were injected i.p. with human insulin (0.75 U/kg for chow-fed mice; 1.5 U/kg for HFD mice) in the presence or absence of DCZ (10 μg/kg), administered either orally or i.p., as indicated. Blood glucose levels were measured immediately before and at specific post-injection time points.

Food intake was measured using single-housed mice for 5 consecutive days by manually weighing food pellets. Food intake during the first 2 days (acclimatization period) was excluded

from the analysis. For refeeding studies, mice were fasted for 24 hours beginning at the onset of the dark cycle, followed by re-feeding with regular chow *ad libitum* for 2 hours.

Indirect calorimetry experiments.

To perform indirect calorimetry and energy expenditure measurements, we used Oxymax-CLAMS chambers (Columbus Instruments) (1, 2). Mice of the indicated genotypes were acclimatized to the chambers for 2 days at 22 °C. On day 3, energy expenditure and related parameters were monitored at 22 °C. On day 4, the temperature was increased to 30 °C (thermoneutrality), and the same measurements were carried out as on day 3. For each mouse, O_2 consumption, CO_2 production, and ambulatory activity (infrared beam breaks) were measured at 13 min intervals. Total energy expenditure (TEE) and respiratory exchange ratio (RER) were calculated based on O_2 consumption and CO_2 production.

STZ-induced diabetes.

To generate a mouse model of diabetes (T2D), 8-week-old male K-GsD mice and control littermates, or WT mice received daily injections of streptozotocin (STZ) (Cat# S0130-50MG, Millipore Sigma) for 5 consecutive days (50 mg/kg i.p.), as described previously (3, 4). Throughout the entire 4-week study, starting from the day of the first STZ injection, the K-GsD mice and control littermates consumed drinking water containing DCZ (10 mg/l). WT mice received 24 nmol/kg i.p. of mouse [D-Ala^2]GIP (Chi Scientific) twice daily at 9 am and 6 pm each day, starting from the day of the first STZ injection.

Treatment with FFAR1 (GPR40), GIP, and GLP1 receptor ligands.

To investigate the metabolic effects of blocking GIP or GLP1 receptor signaling, mice were injected subcutaneously with a monoclonal antibody against the GIP receptor (20 mg/kg; GIPg013; AstraZeneca) (5, 6) or a monoclonal antibody against the GLP1 receptor (19.2 mg/kg; Glp10017; AstraZeneca), respectively (5, 7). Glucose tolerance tests were performed 48 or 24 hours after GIPg013 and Glp10017 treatment, respectively (5). We also treated mice with AM1638 (30 mg/kg, oral administration), a highly selective FFAR1 (GPR40) agonist. Blood was collected immediately before and 30 min after AM1638 administration to determine plasma GIP levels.

Olive oil challenge test.

Mice that had been fasted for 6 hours received an oral gavage of olive oil (10 μl/g body weight, Sigma). Blood was collected before and 60 min after oral oil administration for blood glucose and plasma hormone measurements.

Determination of blood glucose and plasma hormone and metabolite levels.

Blood glucose was measured using an automated blood glucose meter (Glucometer Elite Sensor; Bayer). Blood samples were collected from the tail vein and transferred into EDTA-coated tubes that also contained a dipeptidyl peptidase-4 inhibitor (DPP-4) inhibitor (KR-62436, 0.01 mM). Plasma was obtained by centrifugation of blood at 10,000 x g for 10 min at 4°C. Plasma levels of non-esterified fatty acids (NEFA; Wako Diagnostics), insulin (Crystal Chem #90080), total GIP (Crystal Chem #81527), total GLP1 (Crystal Chem #81508), and glucagon (Crystal Chem #81518) were determined using dedicated kits, according to manufacturers' protocols.

Immunohistochemistry.

Mouse intestinal tissues were fixed overnight in 4% paraformaldehyde and processed using ethanol and xylene, followed by embedding into paraffin blocks. For H&E staining, tissue slices were stained for 10 min in Mayer hematoxylin solution and for 10 sec in eosin Y solution, respectively. For immunofluorescence staining studies, deparaffinized slides were heated in IHC-Tek epitope retrieval buffer (Invitrogen) for 30 min, and blocked and permeabilized in PBS containing 5% goat serum and 0.25% of Triton X-100. Pancreas tissues were incubated overnight at 4°C with rabbit anti-insulin primary antibody (1:1000, #3014, Cell Signaling), whereas intestinal tissues were incubated overnight at 4°C with a mixture of mouse anti-HA primary antibody (1:1000; #2367, Cell Signaling) and rabbit anti-GIP antibody (1:1000; # ab209792, Abcam). After several washing steps, slides were incubated for 1 hour at room temperature with a mix of Alexa Fluor 594- and 488-conjugated secondary antibodies (1:500, ThermoFisher). Images were obtained by using a fluorescent microscope.

To determine the percentage of K-cells (GIP-expressing cells) that co-expressed the hemagglutinin (HA) tag present at the N-terminus of the GsD construct, we analyzed duodenal slices from five different K-GsD mice. The anti-GIP and anti-HA primary antibodies that we used for this analysis are listed in the previous paragraph.

K-cell sorting by FACS, RNA isolation, and qRT-PCR.

To confirm the selective deletion of *Gnas* in K-cells of K-Gs-KO mice, we first washed the isolated mouse duodenum with PBS, cut the tissue into 2 cm pieces in RPMI media containing 0.5 mM EDTA and 2% fetal calf serum, and then stirred for 15 min at 37°C. The cells where then passed through a 70 μm strainer, followed by centrifugation at 37°C for 5 min at 350 x g. K-

cells were sorted from non-K-cells using a biotinylated anti-claudin4 antibody (kindly provided by Dr. Yoko Hamazaki, Kyoto University, Japan), as described previously (8). Total RNA was isolated from sorted K-cells and non-K-cells using TRIzol reagent (Life Technologies). Isolated RNA (100 ng) was used to synthesize cDNA by reverse transcription using a high-capacity cDNA reverse transcription kit (Vazyme, #R333-01). Gene expression analysis was performed by quantitative PCR using the Taq Pro Universal SYBR qPCR Master Mix (Vazyme, Q712-02). Gene expression data were normalized to mRNA levels of *36b4*, a housekeeping gene. The following primers were used: *36b4:* F, ATGGGTACAAGCGCGTCCTG; R: GCCTTGACCTTTTCAGTAAG; *Gnas:* F, ACAAGCAGGTCTACCGGGCCA; R GCCGCCCTCTCCGTTAAACCCA.

Intestinal incretin content.

To measure the intestinal content of GIP and GLP1, the entire mouse intestinal tract was excised from the beginning of the duodenum to the end of the ileum, washed with PBS, weighed, and homogenized mechanically with Ika Ultra-Turrax (Sigma) in an aqueous buffer containing 74% ethanol and 0.15 M HCl, followed by an overnight incubation at 4°C. The homogenate was centrifuged at 12,000 x g for 5 min at 4°C, and the supernatant was diluted 1000-fold for the measurement of GIP and GLP1 concentrations via ELISA (see above).

Pancreatic hormone content.

To study mouse pancreatic glucagon and insulin content, we first measured pancreas weight. Pancreata were then homogenized in acid-ethanol (1.5% HCl in 70% EtOH, 3 ml/pancreas) using a Precellys Evolution Touch homogenizer (Bertin Instruments), followed by a 1 hr

incubation on ice. Tissue homogenates were centrifuged at 10,000 g for 20 min at 4°C, and glucagon and insulin levels were measured in the supernatant using hormone-specific ELISA kits (see above).

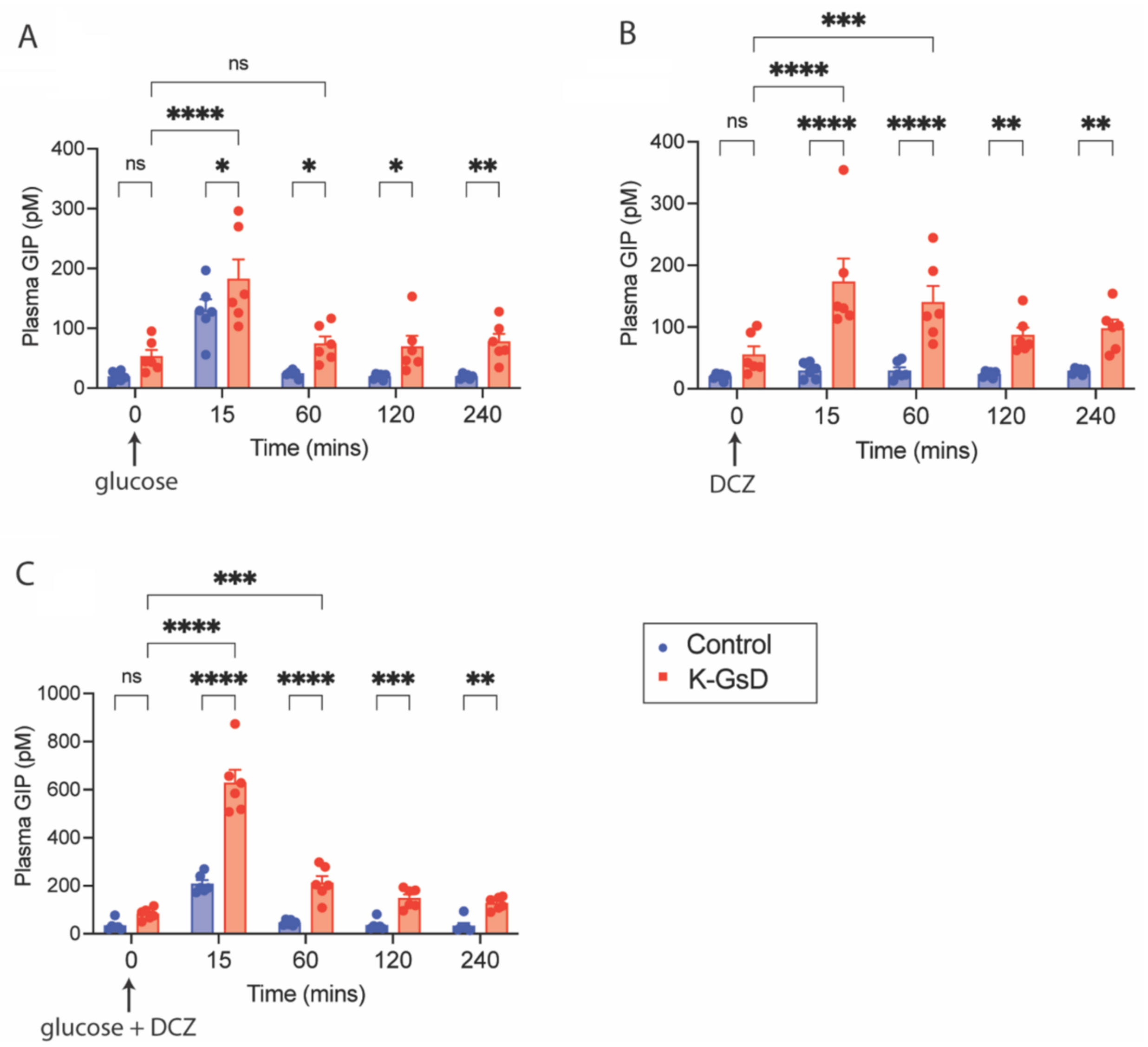

Supplemental Figure 1. Time course and magnitude of GIP release after treatment of K-Gs-D mice with either glucose or DCZ alone or a glucose/DCZ mixture. (A-C) Temporal pattern of plasma GIP levels after different treatments. K-GsD mice and control littermates were treated orally with either glucose (2 g/kg) (**A**) or DCZ alone (10 μg/kg) (**B**), or a glucose/DCZ mixture (**C**). Studies were carried out with 8-10-week-old male mice after a 6-hr fast (diet: regular chow). Data are given as means ± SEM (n = 6 mice/group). *P<0.05, **P<0.01, ***P<0.001, ****P<0.0001; 2-way ANOVA followed by Tukey post-hoc analysis, ns, no statistically significant difference.

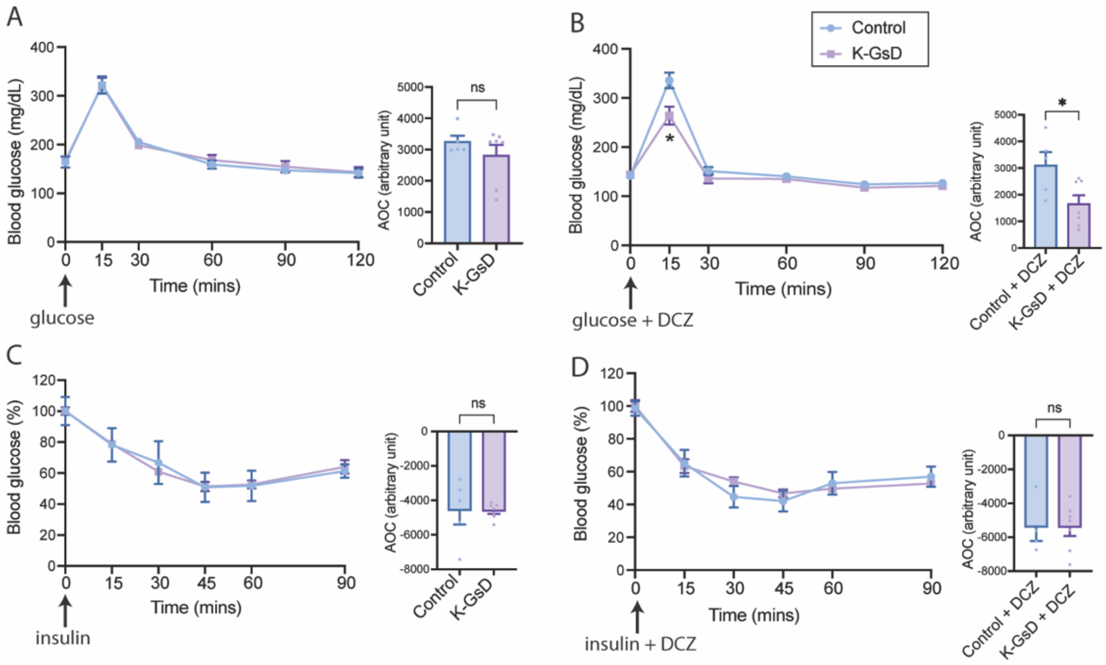

Supplemental Figure 2. **Selective activation of K-cell G$_s$ signaling improves glucose tolerance in female K-GsD mice. (A, B)** Oral glucose tolerance tests (OGTT) carried out with female mice after a 6-hour fast (diet: regular chow). In (**A**), K-GsD mice and control littermates received glucose only (2 g/kg). In (**B**), both groups of mice were treated with oral glucose plus oral DCZ (10 µg/kg). **(C, D)** Insulin tolerance tests (ITT) carried out with female mice after a 4-hour fast. In (**C**), K-GsD and control mice received insulin only **(**0.75 U/kg, i.p.). In (**D**), both groups of mice were co-treated i.p. with insulin plus DCZ (10 µg/kg). AOC values are shown to the right. Data are given as means ± SEM (n = 6 or 7 mice/group). *P<0.05, Student's t-test. ns, no statistically significant difference; AOC, area of the curve.

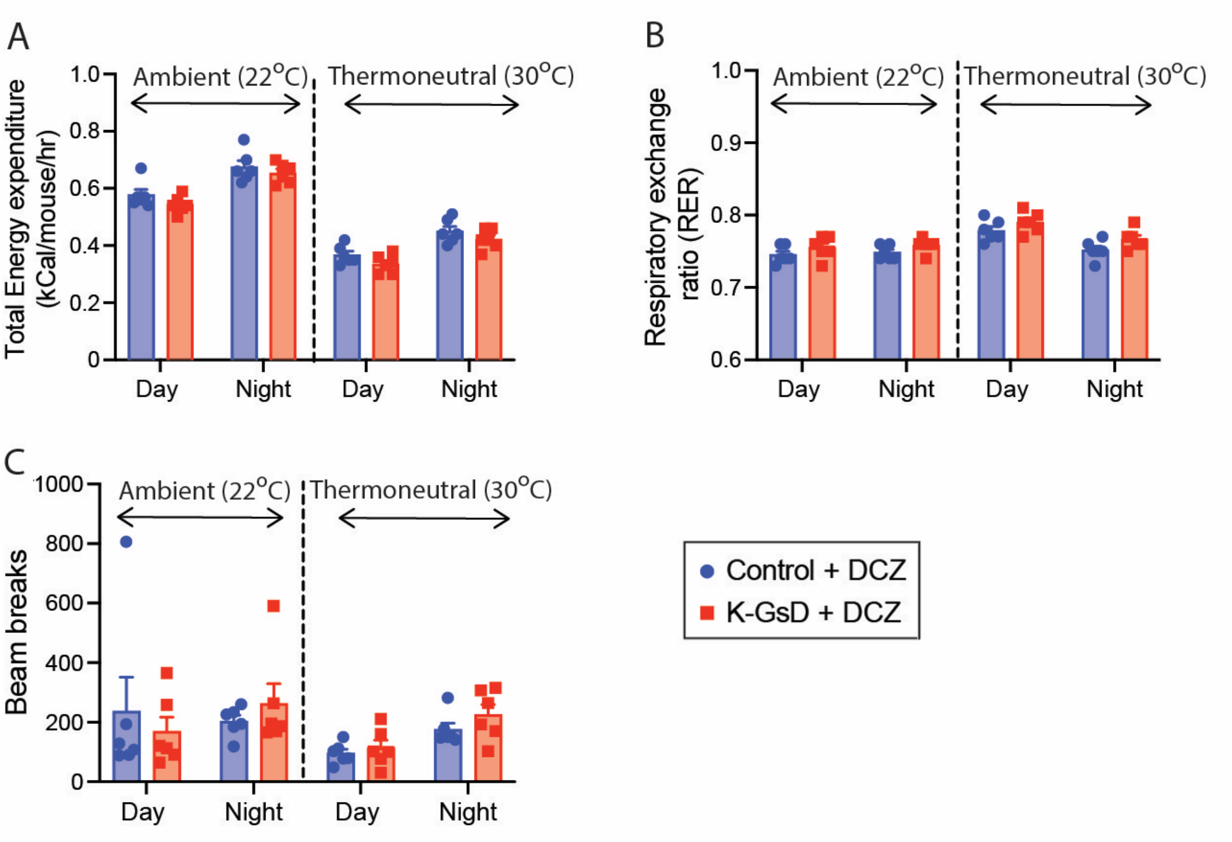

Supplemental Figure 3. Chronic activation of K-cell G_s signaling in obese K-GsD mice has no effect on total energy expenditure, RER, and locomotor activity. (A, B) Indirect calorimetry measurements. Total energy expenditure (**A**), respiratory exchange ratio (**B**), and total ambulatory activity (**C**) of male K-GsD mice and control littermates consuming DCZ drinking water and HFD for 12 weeks. Mice were housed in Oxymax/CLAMS chambers at ambient temperature (22°C) or thermoneutrality (30°C). Mice had free access to food. Data are given as means ± SEM (n = 6 or 7 mice/group).

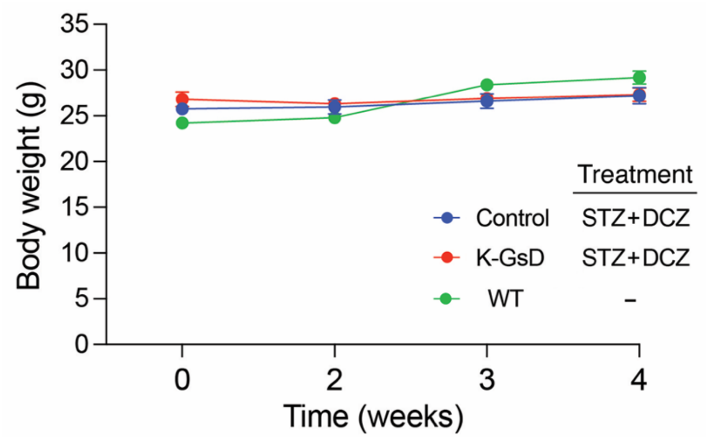

Supplemental Figure 4. Body weight of K-GsD mice and control littermates treated with STZ plus DCZ. K-GsD mice and control littermates (8-week-old males) were treated with STZ for five consecutive days (50 mg/kg i.p. daily). All mice received DCZ via the drinking water (10 mg/l), starting from the first day of STZ treatment, except for WT control mice that received no treatment at all. Data are given as means ± SEM (n= 6-8 mice per group).

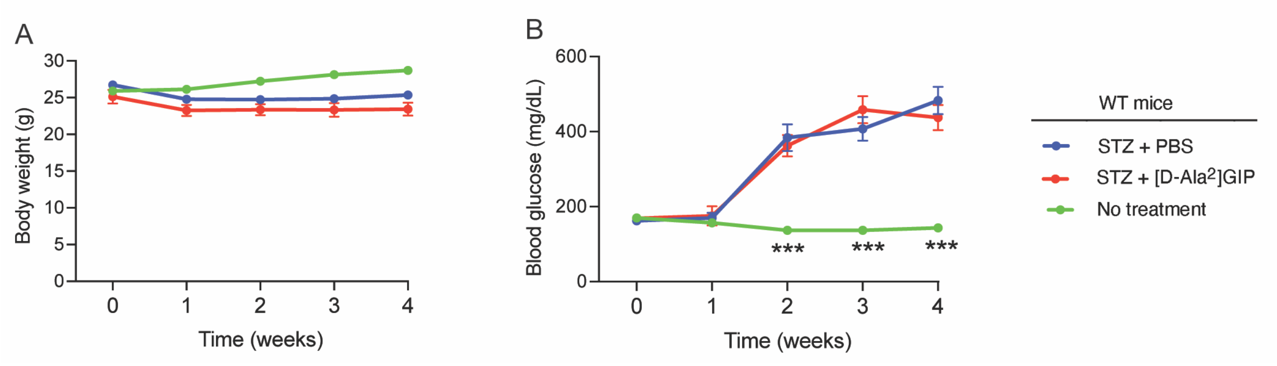

Supplemental Figure 5. Chronic [D-Ala^2]GIP treatment does not affect STZ-induced hyperglycemia in WT mice. (**A, B**) Body weight (**A**) and blood glucose levels (**B**) of WT mice treated with STZ for five consecutive days (50 mg/kg, i.p., daily) and co-treated with either PBS (i.p.) or mouse [D-Ala^2]GIP (24 nmoles/kg, i.p.), a relatively stable GIP analog. Mice were injected with PBS or [D-Ala^2]GIP twice a day (9 am and 6 pm) starting from the first day of STZ treatment, except for WT control mice that received no treatment at all. Treatments were initiated when the mice (males) were 8 weeks old. Data are given as means ± SEM (n= 8 mice per group). ***P<0.001; 2-way ANOVA followed by Tukey post-hoc analysis.

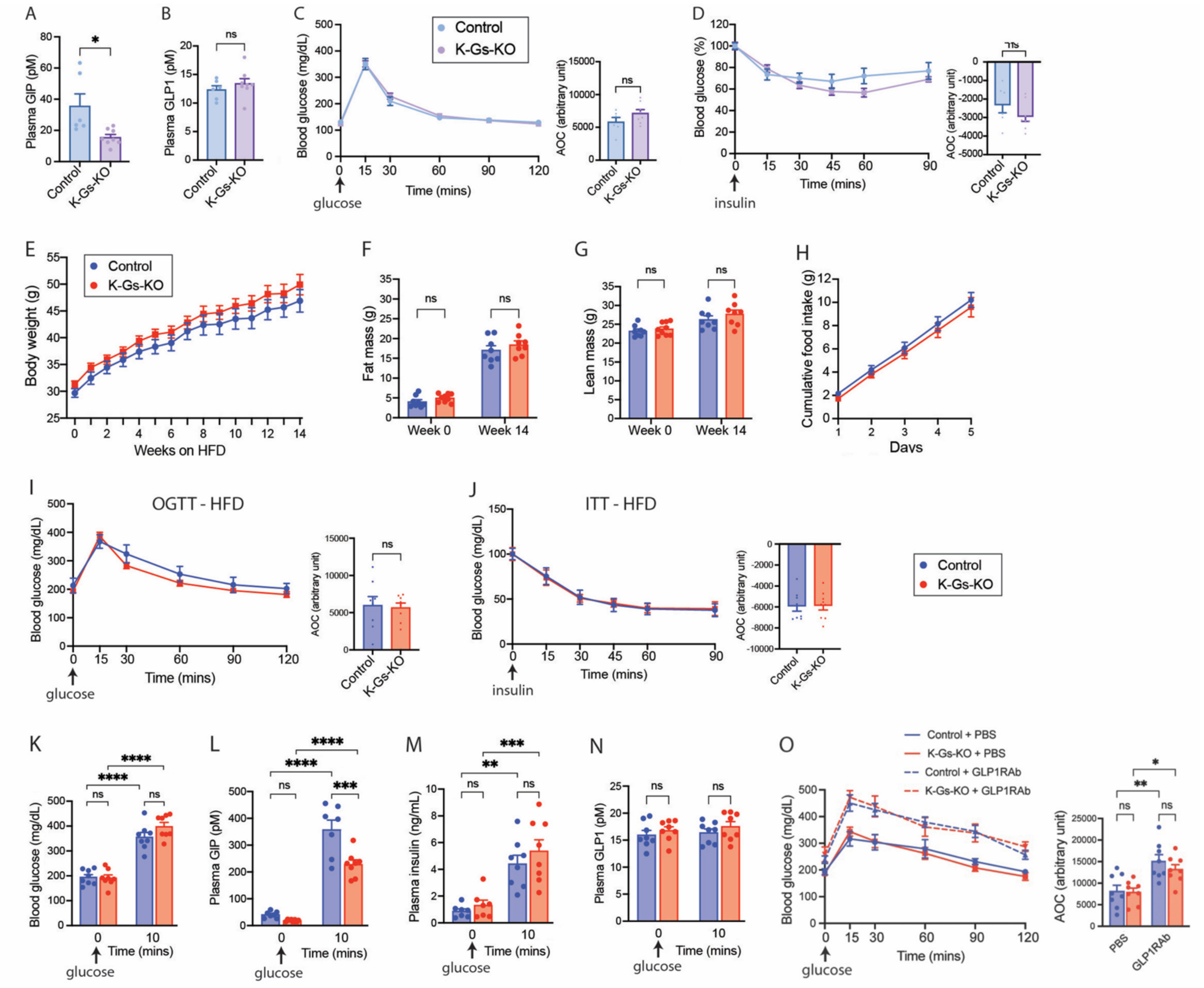

Supplemental Figure 6. Reduced GIP secretion does not affect glucose tolerance in lean female and obese male K-Gs-KO mice. (A, B) Eight-week old female K-Gs-KO mice (diet: regular chow) show reduced plasma GIP levels (**A**), but unchanged plasma GLP1 levels (**B**). Oral glucose tolerance test (2 g/kg) (**C**) and insulin tolerance test (0.75 U/kg, i.p.) (**D**) carried out with female K-Gs-KO and control mice maintained on regular chow. AOC values are shown to the right of each panel. **(I-O)** Metabolic studies with male K-Gs-KO mice and control littermates maintained on a HFD. Metabolic studies were initiated after 8 weeks of HFD feeding. **(E)** Body weight changes during HFD feeding. **(F, G)** Fat mass (**F**) and lean mass (**G**) before and after 14 weeks of HFD feeding. **(H)** Cumulative food intake of single-housed mice. **(I, J)** Oral glucose tolerance test (1 g/kg) (**I**) and insulin tolerance test (1.5 U/kg; i.p.) (**J**). **(K-N)** Blood glucose

levels (**K**), and plasma levels of GIP (**L**), insulin (**M**), and GLP1 (**N**), immediately before (time '0'), and 10 min after oral glucose administration (1 g/kg). **(O)** Oral glucose tolerance test (1 g/kg) carried out with K-Gs-KO mice and control littermates following i.p. pre-treatment with a GLP1 receptor antibody (GLP1RAb; 19.2 mg/kg; Glpr10017) or vehicle (PBS) 24 hr prior to glucose administration. Experiments were performed with mice that had been fasted for 6 hours. Data are given as means ± SEM (n = 6-10 mice/group). *P<0.05, **P<0.01, ***P<0.001, ****P<0.0001; 2-way ANOVA followed by Tukey post-hoc analysis (**F, G, K-N)** or Student's t-test (**A-E, H-J, O)**. ns, no statistically significant difference; AOC, area of the curve.

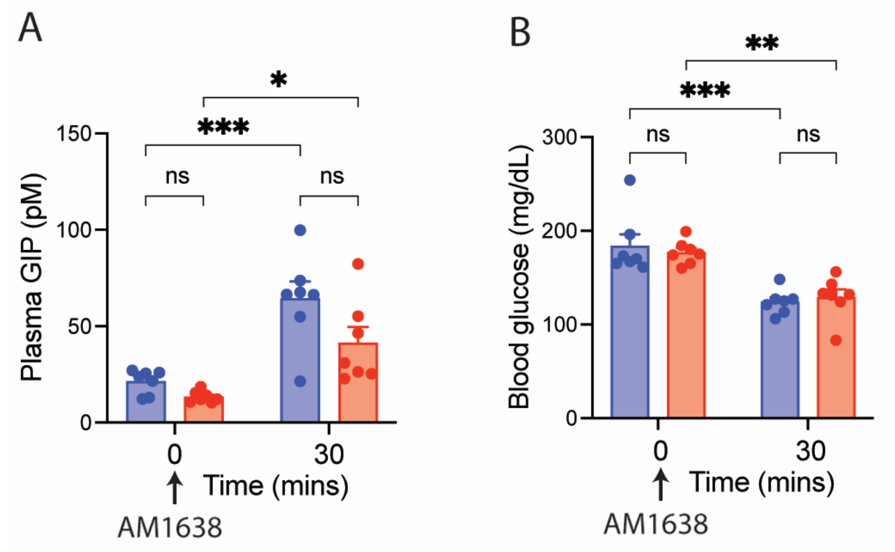

Supplemental Figure 7. AM1638, a selective FFAR1 (GPR40) agonist, enhances GIP secretion and reduces blood glucose in K-Gs-KO mice and control littermates. (**A, B**) Plasma GIP (**A**) and blood glucose (**B**) levels before and 30 min after oral administration of AM1638 (30 mg/kg), a selective FFAR1 agonist. Data are given as means ± SEM (n= 7 male mice per group; age: 10 weeks). *P<0.05, **P<0.01, ***P<0.001, ****P<0.0001; 2-way ANOVA followed by Tukey post-hoc analysis, ns, no statistically significant difference.

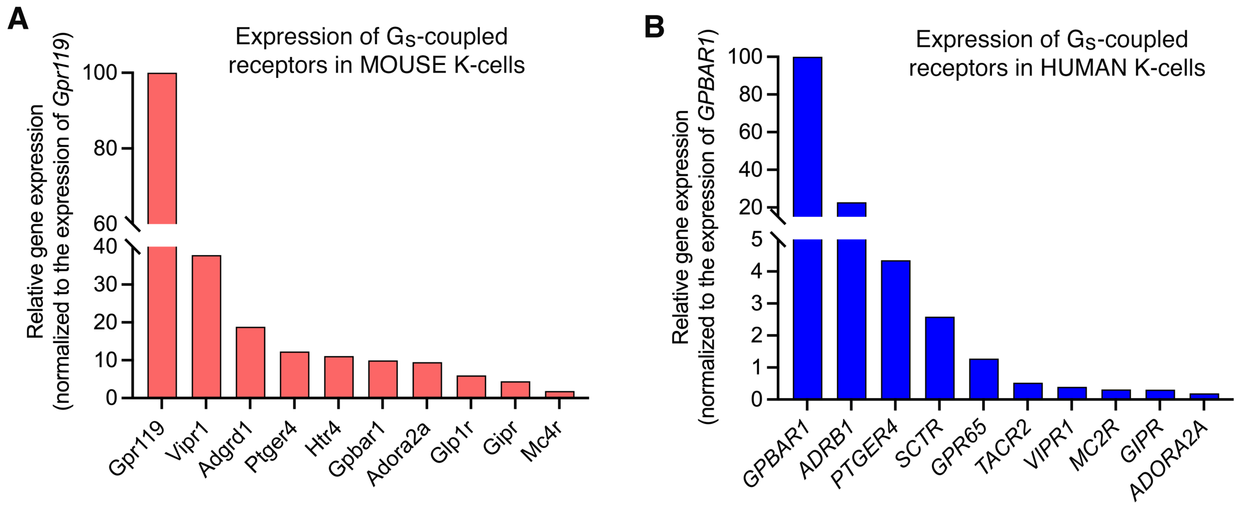

Supplemental Figure 8. Expression of mouse and human K-cell GPCRs that preferentially couple to G_s. (A) Mouse K-cells. Receptor gene expression levels were extracted from single-cell RNA sequencing (scRNAseq) data from GIP-expressing mouse enteroendocrine cells (GSE224223; (9)). **(B)** Human K-cells. Receptor transcript levels were obtained from scRNAseq data from GIP-expressing enteroendocrine cells contained within human duodenal/intestinal organoids (GSE146799; (10)). The ten G_s-coupled receptor genes that are most highly expressed in mouse and human K-cells are shown.

Supplemental Table 1. Primer sequences (mouse) used for PCR genotyping of tail DNA

Gene	Forward primer	Reverse primer
Gip-Cre	5-GCTCCCTCCATTCACTTCACG	5-GTAGTCCCTCACATCCTCAGG
$Gnas^{flox/flox}$	5-TTCGGTCTCGTCCCCTTAGTTG	5-AACAAATCGCACACCCCAGTGAGG
LSL-GsD	5-CTCGAAGTACTCGGCGTAGG	5-CTTGGCAATCCGGTACTGTT

Supplemental References

1. Gautam D, Gavrilova O, Jeon J, Pack S, Jou W, Cui Y, et al. Beneficial metabolic effects of M3 muscarinic acetylcholine receptor deficiency. *Cell Metab.* 2006;4(5):363-75.
2. Tanaka N, Takahashi S, Matsubara T, Jiang C, Sakamoto W, Chanturiya T, et al. Adipocyte-specific disruption of fat-specific protein 27 causes hepatosteatosis and insulin resistance in high-fat diet-fed mice. *J Biol Chem.* 2015;290(5):3092-105.
3. Furman BL. Streptozotocin-Induced Diabetic Models in Mice and Rats. *Curr Protoc.* 2021;1(4):e78.
4. Jain S, Ruiz de Azua I, Lu H, White MF, Guettier JM, and Wess J. Chronic activation of a designer G(q)-coupled receptor improves beta cell function. *J Clin Invest.* 2013;123(4):1750-62.
5. Svendsen B, Capozzi ME, Nui J, Hannou SA, Finan B, Naylor J, et al. Pharmacological antagonism of the incretin system protects against diet-induced obesity. *Mol Metab.* 2020;32:44-55.
6. Ravn P, Madhurantakam C, Kunze S, Matthews E, Priest C, O'Brien S, et al. Structural and pharmacological characterization of novel potent and selective monoclonal antibody antagonists of glucose-dependent insulinotropic polypeptide receptor. *J Biol Chem.* 2013;288(27):19760-72.

7. Biggs EK, Liang L, Naylor J, Madalli S, Collier R, Coghlan MP, et al. Development and characterisation of a novel glucagon like peptide-1 receptor antibody. *Diabetologia.* 2018;61(3):711-21.
8. Nagatake T, Fujita H, Minato N, and Hamazaki Y. Enteroendocrine cells are specifically marked by cell surface expression of claudin-4 in mouse small intestine. *PLoS One.* 2014;9(6):e90638.
9. Hayashi M, Kaye JA, Douglas ER, Joshi NR, Gribble FM, Reimann F, et al. Enteroendocrine cell lineages that differentially control feeding and gut motility. *Elife.* 2023;12.
10. Beumer J, Puschhof J, Bauzá-Martinez J, Martínez-Silgado A, Elmentaite R, James KR, et al. High-Resolution mRNA and Secretome Atlas of Human Enteroendocrine Cells. *Cell.* 2020;182(4):1062-4.
